# Tumor Extracellular Vesicles Regulate Macrophage-Driven Metastasis through CCL5

**DOI:** 10.3390/cancers13143459

**Published:** 2021-07-10

**Authors:** Daniel C. Rabe, Nykia D. Walker, Felicia D. Rustandy, Jessica Wallace, Jiyoung Lee, Shannon L. Stott, Marsha Rich Rosner

**Affiliations:** 1 Ben May Department for Cancer Research, University of Chicago, Chicago, IL 60615, USA; drabe@mgh.harvard.edu (D.C.R.); nykia.d.walker@gmail.com (N.D.W.); feliciarustandy@gmail.com (F.D.R.); jiyounglee@gwu.edu (J.L.); 2 Center for Cancer Research, Massachusetts General Hospital, Boston, MA 02129, USA; 3 BioMEMS Resource Center, Center for Engineering in Medicine and Surgical Services, Massachusetts General Hospital and Harvard Medical School, Boston, MA 02129, USA; jessica.wallace.100@comcast.net; 4 Broad Institute of MIT and Harvard, Cambridge, MA 02142, USA

**Keywords:** metastasis, tumor-associated macrophages (TAMs), extracellular vesicles (EVs), exosomes, CCL5, tumor microenvironment (TME), triple-negative breast cancer (TNBC)

## Abstract

**Simple Summary:**

About 10–20 percent of patients with breast cancer are diagnosed with triple-negative breast cancer (TNBC). These tumors are named for their lack of expression of estrogen receptor (ER), progesterone receptor (PR), and amplification of human epidermal growth factor receptor 2 (HER2). These genes are targeted by therapies in other breast cancer patients. However, most TNBC patients recur within 5 years. Understanding how and why these tumors metastasize will help clinicians better treat these underserved cancer patients. TNBC tumors are highly infiltrated by tumor-associated macrophages (TAMs) that promote tumorigenesis and metastasis. Our study elucidates how the tumor co-opts macrophages recruited to the tumor through extracellular vesicles (EVs), further increasing tumor metastasis. Expression of tumor CCL5 regulates EV secretion and cargo that further alters macrophage phenotype to drive tumor metastasis. Together, our data suggest a more extensive role of EVs in the biology of tumor metastasis as well as their potential use as biomarkers.

**Abstract:**

Purpose: To understand how tumor cells alter macrophage biology once they are recruited to triple-negative breast cancer (TNBC) tumors by CCL5. Method: Mouse bone marrow derived macrophage (BMDMs) were isolated and treated with recombinant CCL5 protein alone, with tumor cell conditioned media, or with tumor extracellular vesicles (EVs). Media from these tumor EV-educated macrophages (TEMs) was then used to determine how these macrophages affect TNBC invasion. To understand the mechanism, we assayed the cytokine secretion from these macrophages to determine how they impact tumor cell invasion. Tumor CCL5 expression was varied in tumors to determine its role in regulating macrophage biology through EVs. Results: Tumor EVs are a necessary component for programming naïve macrophages toward a pro-metastatic phenotype. CCL5 expression in the tumor cells regulates both EV biogenesis/secretion/cargo and macrophage EV-education toward a pro-metastatic phenotype. Analysis of the tumor EV-educated macrophages (TEMs) showed secretion of a variety of factors including CXCL1, CTLA-4, IFNG, OPN, HGF, TGFB, and CCL19 capable of remodeling the surrounding tumor stroma and immune infiltrate. Injection of tumor cells with macrophages educated by metastatic tumor cell EVs into mice increased tumor metastasis to the lung. Conclusion: These results demonstrate that tumor-derived EVs are key mediators of macrophage education and likely play a more complex role in modulating tumor therapeutic response by regulating the tumor immune infiltrate.

## 1. Introduction

Of the women in the United States that develop breast cancer, 15–20% will have basal-like or triple-negative breast cancer (TNBC) [1]. Triple-negative tumors lack expression of estrogen receptor (ER), progesterone receptor (PR), and amplification of human epidermal receptor 2 (HER2) [1]. Clinical outcomes have improved for many patients with breast cancer, as high as 98% five-year survival rates in patients treated with targeted anti-ER or anti-HER2 therapies. However, patients with TNBC have five-year survival rates close to 24% [2], and this disproportionately affects African-American women and lower income women [1,3]. Thus, it is important to identify mechanisms that drive metastatic progression of TNBCs as well as potential targets for therapeutic treatment and biomarkers for response to therapy or progression.

Tumor cells are driven to metastasize in part through interaction with cells in the microenvironment. Tumor-associated macrophages (TAMs), generally characterized as alternatively activated macrophages, play an essential role in driving metastasis [4,5]. Previous studies showed that deleting Colony Stimulating Factor-1 (CSF-1/M-CSF) in the mouse mammary tumor virus–polyoma virus middle T antigen breast cancer mouse model (MMTV-PyMT) delayed metastatic tumor progression without altering mammary tumor growth [6]. Furthermore, liver cancer studies demonstrated that macrophage ablation enhanced the anti-tumor effects of targeted therapies [7]. Finally, the extent of TAM infiltration, determined by Cluster of Differentiation 163 (CD163) staining, correlates positively with TNBC tumors, negatively with less aggressive ER+ tumors, and positively with poor outcome within TNBC tumors [8]. Thus, pro-metastatic TAMs play a significant role in the progression of TNBC tumors and contribute to poor prognosis of patients.

TAMs are recruited to mammary tumors through induction of a variety of cytokines and chemokines including C-C Motif Chemokine Ligand 2 (CCL2), CCL3, CCL5, Colony Stimulating Facotor-1 (CSF-1/M-CSF), and Granulocyte-Macrophage Colony-Stimulating Factor (GM-CSF or CSF2) [9,10,11,12,13]. This interaction is further complicated by proteomic studies that identified multiple subsets of cytokine-secreting TAMs in vivo [14]. A number of studies have implicated CCL5 [10,11] in the recruitment of macrophages to mammary tumors. Our previous study showed that CCL5 mediates macrophage recruitment to TNBCs, and the metastasis suppressor Raf Kinase Inhibitory Protein (RKIP) blocks this recruitment by inhibiting CCL5 expression in tumor cells and CCR5 expression in macrophages [11]. CCL5 has also been shown to play a role in the recruitment of T cells. Specifically, studies have shown that tumor-derived CCL5 can recruit T-regulatory cells (T-regs) into the tumor, leading to CD8+ T-cell apoptosis. Blockade of CCL5 leads to decreased tumor growth in immune competent models, suggesting that T-cells are required to reduce tumor bulk [15]. Additionally, stromal CCL5 and tumor CCR3 (but not CCR1 or CCR5) expression together are associated with poor outcome in TNBC patients [16]. Although factors such as CCL5 that enable recruitment of TAMs to mammary tumors have been identified, the process by which recruited macrophages are educated or polarized by tumor cells is not well-understood.

One possible mechanism connecting tumor and macrophage education is through EVs secreted from the tumor cells and taken up by the immune system. EVs are small micro-vesicles with a double lipid bilayer, secreted by most cell types, and of endocytic or membrane origin [17]. Small EVs (which include exosomes) are often between 40 to 150 nm and thought to be formed through invagination of the endosome membrane to form multi-vesicular bodies (MVBs) [18,19,20]. Alternative pathways of secretion for large EVs are thought to occur through exocytosis at the plasma membrane. EV markers include Alix1, CD9, CD63, CD81, and TSG-101 [17,21]. Knock-down screens of genes involved in EV secretion identified Rab27a as necessary for full secretion of EVs from cells. Rab27a is required for docking of MVBs at the plasma membrane [22]. The ESCRT complex additionally assists in MVB sorting to the membrane before being assisted by Rab proteins to dock and fuse with the plasma membrane via SNARE-mediated membrane fusion to release exosomes into the extra-cellular space [23]. The most widely used protein to block EV secretion in cells for in vivo studies is Rab27a [17,22].

A number of studies suggest that small EVs, including exosomes, modulate stromal and immune response within the tumor microenvironment [24,25]. Tumor-derived EVs promote formation of pre-metastatic niches [20,25] and drive progenitor cells within the bone marrow toward a pro-metastatic state [26]. Tumor-derived EVs stimulate myofibroblast differentiation [27], suppress immune response by impeding T cell activation [28], inhibit NK function [29], or potentiate regulatory T cell (T-reg) activity [30]. Stromal EVs induce tumor cell migration [31] and can mediate chemotherapy resistance in breast cancer cells [32]. EVs can also be taken up by macrophages, likely through clathrin-dependent phagocytosis [33,34,35]. However, direct evidence that EVs regulate naïve macrophage polarization, particularly in primary tumors, or their functional effect on macrophage-driven metastasis in vivo is lacking.

Our results indicate that tumor EVs alone can educate macrophages to a TAM phenotype that drives invasion and metastasis of TNBC. The absence of EVs, either through removal by ultracentrifugation or reduced secretion through Rab27a knockdown reduce the ability of tumors to program macrophages. Furthermore, we show that macrophage education occurs through an indirect mechanism involving stimulation of tumor cells by CCL5. CCL5 expression within the tumor increases the secretion of tumor EVs and alters the ability of EVs to educate macrophages. This results in TAMs that reflect the metastatic state of the tumor cells. Clinically, the genes we identified in this study are highly correlated to CCL5, and cluster in a subset of TNBC patient tumors within The Cancer Genome Atlas (TCGA) cohort. Taken together, these results indicate that tumor EVs play an integral role in education of non-resident macrophages to a pro-metastatic TAM phenotype in TNBCs, driving tumor metastasis.

## 2. Materials and Methods

### 2.1. Cell Culture

MDA-MB-231, 293T, and L929 were obtained from ATCC. MDA-MB-231 1833 [36] (referred to as BM1) cells were obtained from Andy Minn and E0771-LMB cells were obtained from Robin Anderson [37]. Numerous vials were frozen upon original receipt of the cells, and all work was done within 15 passages of the initially received lines. Late passage cells were sent to Idexx for cell line authentication using STR analysis and cells were routinely checked for mycoplasma contamination (Lonza, Basel, Switzerland). BM1, 293T, and LMB cell lines were cultured in high-glucose DMEM media with L-glutamine (Corning, Corning, NY, USA) supplemented with 10% fetal bovine serum (Corning), and 50 U/mL penicillin and 50 μg/mL streptomycin (Gibco, Dublin, Ireland) and L929 grown in RPMI 1640 (Corning) with the same supplementation as above.

### 2.2. Lentiviral Transductions

All lentiviral work was done according to institutional biosafety rules, utilizing BSL3 practices and performed in a BSL2 hood. One million 293T cells were plated in a T-25 flask the evening prior. The following day, lentiviral vectors were incubated with 4th generation viral packaging vectors and Transit LT-1 for 30 min as described by the manufacturer’s protocol. DNA/LT-1 mixtures were then used to transfect 293T cells for viral production. Transfected cells were grown for 24–48 h prior to viral harvesting. After incubation, viral containing media was removed, centrifuged at 2000× *g* to removed dead cells and debris, then filtered through a 0.45 μm PES syringe filter. Polybrene was added to media for a final concentration of 8 ng/mL. Media was then added to target cells. Following a 24-h transduction period, cells were washed, trypsinized, and plated.

### 2.3. shRNA Work

Lentiviral shRNA plasmids targeting Rab27a from The RNAi consortium (TRC) were purchased from Open Biosystems. Lentiviruses were produced as described above. BM1 cells were transduced and selected in 3 μg/mL of puromycin and LMB cells with 10 μg/mL of puromycin for 14 days. Five shRNA targets against Rab27a were used for both human and mouse Rab27a, with a non-targeting shRNA used as control.

### 2.4. CRISPR-Cas9 Work

Lentiviral all-in-one plasmids containing Cas9 as well as guide RNAs were purchased from Applied Biological Materials. Lentivirus was produced as described above. BM1 cells were then infected, then selected for 14 days using 3 μg/mL of puromycin. Transduced cells were assayed for CCL5 expression using a CCL5 ELISA from Ray Biotech as well as by qPCR. Media samples were concentrated 10× to ensure even low levels in KO cells could be measured.

For dCas9-KRAB knock-down and dCas9-VPR over-expression of *Ccl5* in mouse LMB cells, sequences were determined using Broad’s online sgRNA tool, and sgRNA sequences were ordered from IDT and inserted into sgRNA vectors obtained from Broad. Cells were first transduced with dCas9-KRAB or dCas9-VPR and selected with 10 μg/mL blasticidin followed by sgRNA transduction and selection with 10 μg/mL puromycin for 14 days each. RNA levels of *Ccl5* were measured in cells and EVs using ddPCR with *Gapdh* as a loading control.

### 2.5. qRT-PCR Primers

Hs GAPDH-F: TGCACCACCACCTGCTTAGC
Hs GAPDH-R: GGCATGGACTGTGGTCATGAG
Hs CCL5-F: CCAGCAGTCGTCTTTGTCAC
Hs CCL5-R: CTCTGGGTTGGCACACACTT
Mm Ccl5-F: TTTGCCTACCTCTCCCTCG
Mm Ccl5-R: CGACTGCAAGATTGGAGCACT
Mm Slpi-F: GGCCTTTTACCTTTCACGGTG
Mm Slpi-R: TACGGCATTGTGGCTTCTCAA
Mm Mmp12-F: CTGCTCCCATGAATGACAGTG
Mm Mmp12-R: AGTTGCTTCTAGCCCAAAGAAC
Mm Ccl7-F: GCTGCTTTCAGCATCCAAGTG
Mm Ccl7-R: CCAGGGACACCGACTACTG
Mm Tnfr2-F: ACACCCTACAAACCGGAACC
Mm Tnfr2-R: AGCCTTCCTGTCATAGTATTCCT
Mm Grn-F: ATGTGGGTCCTGATGAGCTG
Mm Grn-R: GCTCGTTATTCTAGGCCATGTG
Hs Grn F: ATCTTTACCGTCTCAGGGACTT
Hs Grn R: CCATCGACCATAACACAGCAC
Hs TNFR2 F: CGGGCCAACATGCAAAAGTC
Hs TNFR2 R: CAGATGCGGTTCTGTTCCC
Hs CCL5 Sequencing F: TTAGGGGATGCCCCTCAACT
Hs CCL5 Sequencing R: CTGAGACTCACACGACTGCTG
Hs Rab27a-F: GCTTTGGGAGACTCTGGT
Hs Rab27a-R: TCAATGCCCACTGTTGTGATAAA
Mm Rab27a-F: TCGGATGGAGATTACGATTACCT
Mm Rab27a-R: TTTTCCCTGAAATCAATGCCCA

mCCL5i sgRNA targets
AGAGATCTTCATGGTACCCG
GTACCCGCGGCAGAGGCTGT
ATCTCCCACAGCCTCTGCCG
CTTCATGGTACCCGCGGCAG
GGCAGCTGCAGAGATCTTCA

mCCL5a sgRNA targets
TTATGACAGCAACAAGTGTT
TGGAAACTCCCCAAGTCCTG
GTGGAAACTCCCCAAGTCCT
TGTGGAAACTCCCCAAGTCC
CCCCCCAGCCCCAGGACTTG

### 2.6. Antibodies, Cytokine Arrays, ELISAs

RKIP (derived in lab from serum of rabbits exposed to an RKIP peptide), Rabbit anti-CD63 antibody (System Biosciences, Palo Alto, CA, USA), Rab27a (AF7245, R&D Systems, Minneapolis, MN, USA), F4/80 (MCA497GA, AbD Serotec, Bio-Rad, Hercules, CA, USA), Foxp3 clone:FJK-16s (13-5773, eBioscience, San Diego, CA, USA), FITC-CD63 (cat # 550759, BD, Franklin Lakes, NJ, USA), CD9-PerCP-Cy 5.5 (561329, BD), CD81-Pacific Blue (349515, BD), Alix Ab (3A9) (65678, Novus, Centennial CO, USA), APC-Fire IgG (406623, BioLegend, San Diego, CA, USA), Exosome-human CD63 isolation/detection reagent (10606D, Life Technologies, Waltham, MA, USA), CCL5 ELISA (ELH-RANTES-1, Ray Biotech, Peachtree Corners, GA, USA), Mouse Cytokine Array (L308, Ray Biotech)

### 2.7. Tumor Growth Assays

Two thousand tumor cells were plated in 200 µL of media per well in a 96-well culture plate (Falcon, Corning, NY, USA). Six replicates per condition were used for all assays. Cells were then monitored using an Incucyte Zoom (Essen Bioscience, Sartorius, Ann Arbor, MI, USA), with 20× phase-contrast images taken every four hours. Percent confluence was calculated using Incucyte Analysis Software and exported as a CSV file for either individual time and data points or average data points per time point. Exported data was graphed as individual points (when available) using Graph Pad Prism and statistical analysis performed as described below.

### 2.8. Tumor Migration Assays

20,000 tumor cells were plated in 200 µL of media per well in a 96-well culture plate (Falcon) and allowed to adhere overnight. Six replicates per condition were used for all assays. Each well was precisely scratched using an Incucyte Woundmaker Tool (Essen Bioscience, Sartorius). Wells were washed to remove scratched cells and debris, and 200 µL of fresh media per well was added. Cells were then monitored using an Incucyte Zoom (Essen Bioscience, Sartorius), with 20× phase-contrast images taken every four hours. Migration was calculated as (%Wound Healing) using the Incucyte Scratch Wound Analysis Software Module and exported as a CSV file for either individual time and data points or average data points per time point. Exported data was graphed as individual points (when available) using Graph Pad Prism and statistical analysis performed as described below.

### 2.9. Invasion Assays

As previously described, 2 × 10^4^ BM1 cells or 1 × 10^5^ E0771-LMB cells were plated in 24-well trans-well inserts with 8 μm pores (Corning, 353097) coated with growth factor depleted basement membrane extract (Trevigen, Gaithersburg, MD, USA) [11,38]. After incubating at 37 °C for 24 h, inserts were transferred to an empty well and stained with 4 ng/μL of Calcein AM (Corning) for one hour. Stained cells were gently wiped with Q-tips to remove cells on the top layer of the insert, then placed in non-enzymatic dissociation solution (Trevigen) using gentle shaking for one hour at 37 °C and 150 RMP. Fluorescence was measured using a Victor X3 fluorescent plate reader with excitation at 465 nm and emission at 535 nm.

### 2.10. Tumor EV Isolations

Tumor EV isolations were done in accordance with MISEV2018 guidelines [39]. For all experiments, tumor conditioned media (CM) was spun at 2000× *g* for 10 min to remove cells and cell debris, and then filtered through a 0.22 μm PES syringe filter (Millipore). For ultracentrifugation experiments, prepared CM was spun at 100,000× *g* for 70 min in polycarbonate, hard-wall tubes. EV cleared CM was then removed and saved for experiments. Remaining media was removed and EV pellets were resuspended in the same starting volume of serum free media from which they were enriched.

For Izon qEV columns CM from four 15-cm plates was prepared as above, followed by concentration using an Amicon with a 3 kD pore (MilliporeSigma, Darmstadt, Germany) to 500 μL. Concentrated media was loaded onto a rinsed qEV column. Fractions 1–6 (column dead volume, 3 mL) were collected in one tube, followed by collection of EVs in fractions 7–9 (1.5 mL). Following isolation, columns were cleaned with 750 μL of 0.5 M NaOH followed by washing with 30 mL of PBS (Corning) before re-use.

### 2.11. SEM Measurements of EVs

Copper grids were glow discharged for 30 s, stained with 3.5 μL of sample for 1 m, rinsed twice with 1% PTA, and then stained with 1% PTA for 45s. Grids were imaged on an FEI Technai G2 F30 300kV transmission electron microscope.

### 2.12. Nano Tracking Analysis (NTA) Utilizing the Malvern NS300

Enriched EV samples were diluted 1:20 in PBS. 1 mL of diluted EVs were then used for NTA. Once the flow cell and tubing were primed with the sample, the syringe pump was set to a flow rate of 50 μL/s. Three videos of 45 s were recorded of enriched EVs. The videos were processed according to standard Malvern protocols and then exported.

### 2.13. Flow Cytometry

We collected conditioned media from cells plated at 1 × 10^6^ cells and EVs were isolated as described above using qEV original. Next, we isolated exosomes from other EVs using a human CD63 Dynabeads (Invitrogen, Waltham, MA, USA), per the company’s protocol for use in flow cytometry. The exosomes conjugated with Dynabeads were washed in 300 μL of Dynabead’s isolation buffer. One hundred microliters of exosomes were stained in separate polystyrene tubes with 0.1–10 μg/mL of Alix1, CD9, or CD63 (each antibody was measured individually) and were incubated for 60 min at room temperature. The exosomes were washed three times using 300 μL of isolation buffer and then placed on magnetic stand and discard the supernatant. This step was repeated once and resuspended in 500 μL of PBS with 0.02% sodium azide. The exosomes were kept in foil and immediately analyzed using a BD Fortessa 4–15. Each antibody was measured independently, so no compensation was needed. Unstained and isotype controls were measured to establish a baseline for gating negative particles. Analysis of flow data and graphs were produced using FlowJo.

### 2.14. Tumor Educated Macrophage Programming

Bone marrow was isolated from the femur and tibia of 6–10-week-old C57Bl/6 mice (Charles Rivers). Red cell lysis buffer (Santa Cruz) was used to remove red blood cells. Remaining bone marrow was counted, and 1 million cells were plated per well in a 6-well plate and cultured in 70% RPMI 1640 supplemented with 10% FBS (Corning) and 50 U/mL penicillin, and 50 μg/mL streptomycin (Invitrogen) mixed with 30% L929 Conditioned Media. Media was replenished at days two and six during culture. On day seven, bone-marrow derived macrophages (BMDMs) were washed 2–3 times with PBS (Corning) and then treated with 20 ng/mL of mouse M-CSF spiked into tumor cell conditioned media, serum free media containing tumor EVs, or serum free media as a negative control. EV size and numbers were determined after isolation using nanoSight. 1 × 10^8^ EVs/mL were used for programming with BM1 tumor EVs or 1 × 10^9^ EVs/mL for LMB EVs.

For TEMs, after programming, cells were washed with PBS 2-3 times. Then 1 mL of serum free DMEM was added. After incubation at 37 °C for 24 h, conditioned media was removed and cells and cell debris were removed by centrifugation at 2000× *g* for 10 min, and immediately used to treat TNBC cells. TEMs were lysed with TRIzol and RNA extracted using Direct-zol kit (Zymo Research, Irvine, CA, USA) per manufacturers protocol.

### 2.15. Tumor Associated Macrophage Isolations

As previously described [11], tumors were grown to approximately 0.75 g before being harvested. Tumors were dissociated both physically with scissors to 1–2 mm pieces and using C-tubes and a gentleMACS dissociator (Miltenyi Biotech, Bergisch Gladbach, Germany) as well as enzymatically using the human tumor dissociation kit (Miltenyi Biotech). Cells were filtered through a 70 μm mesh filter. Mononuclear cells were isolated using Ficoll-Paque PREMIUM (GE Healthcare, Chicago, IL, USA) gradient centrifugation at 420 RPM for 45 min. Macrophages were then obtained using CD11b positive selection beads (Miltenyi Biotech).

For tumor derived macrophages, 1 × 10^6^ TAMs were plated in one well of a 6-well plate. After 30 min, cells were washed with PBS to ensure only viable macrophages attached to the plate remained. Cells were incubated for 24 h to obtain conditioned media in serum free DMEM. Cells and cell debris were removed by centrifugation at 2000× *g* for 10 min prior to use in subsequent assays.

### 2.16. Conditioned Media

For TEM conditioned media, cells were washed twice with PBS and once with serum free DMEM after 48 h of programming with CM or EVs. Each well was then incubated in serum free DMEM for 24 h to collect TEM CM. TEM CM was then spun at 2000× *g* for 10 min to remove cells and cell debris.

For tumor derived macrophages, 5 × 10^5^ TAMs were plated in one well of a 6-well plate. After 30 min, cells were washed with PBS to ensure only viable macrophages attached to the plate remained. Cells were incubated for 24 h to obtain conditioned media in serum free DMEM. Cells and cell debris were removed by centrifugation at 2000× *g* for 10 min.

### 2.17. Mice

All mice were housed and handled according to the University of Chicago Institutional Animal Care and Use Committee guidelines. Athymic nude, Balb/c mice, and C57Bl/6 were purchased from Charles Rivers. Mice were injected with 2 × 10^6^ human or 5 × 10^5^ mouse tumor cells in 100 μL of DPBS into the fat pad of 5–6-week-old female mice. Tumor volumes were measured twice per week and calculated as volume = (π/6) × width^2^ × length, where width is the shorter of the two distances. Tumors were grown to ~1000 cm^3^ (or ~0.75–1 g) and removed for analysis.

For LMB co-injection studies, TEMs were injected into the fat pad of C57Bl/6 mice mixed with E0771-LMB cells at a ratio of 1 TEM:1 tumor cell. Lung metastases were assayed at the end of the study by fixing in formalin and sectioning. Six 5 μm sections were quantified for number and size of metastases. Sections were 100 μm apart, and number of metastases counted were added to give number of metastases per lung.

For BM1 co-injection studies, TAMs isolated from BM1 tumors in mice were immediately injected into the fat pad of nude mice mixed with MDA-MB-231 tumor cells at a ratio of 1 TAM: 2 Tumor cells.

### 2.18. Immunohistochemistry

All immunohistochemistry was performed at the University of Chicago Human Tissue Resource Center. Tissue sections were deparaffinized and rehydrated through xylenes and serial dilutions of EtOH to deionized water. For F4/80, samples were incubated in antigen retrieval buffer (S1699, DAKO, Agilent, Santa Clara, CA, USA) and heated in a steamer at 97 ℃ for 20 min. Anti-mouse F4/80 antibody (1:200, MCA497GA, AbD Serotec) was applied on tissue sections for 1 h at room temperature. Following staining and TBS wash, tissue sections were incubated with biotinylated anti-rat IgG (10 μg/mL, BA-4001, Vector laboratories) for 30 min at room temperature. The antigen-antibody binding was detected by Elite kit (PK-6100, Vector Laboratories) and DAB (DAKO, K3468) system according to the manufacturers’ protocols. Following staining, tissue sections were briefly immersed in hematoxylin for counterstaining and were covered with cover glasses. Stained tissue sections were scanned at 20× magnification and analyzed using Aperio Imagescope ePathology software. To quantify infiltrating macrophages, number of F4/80 positive cells were counted using Imagescope and normalized to the area imaged.

### 2.19. TCGA Analysis

All analysis of human tumor samples from the cancer genome atlas (TCGA) were done using cbioportal.org. *z*-score normalized mRNA expression was compared for the 1100 samples from the Breast Invasive Carcinoma (TCGA, Firehose Legacy) patient set with mRNA expression from RNAseq. Heatmaps and clinical data were ordered using heatmap clustering order (hierarchical clustering done by cBioPortal) and exported.

### 2.20. Statistical Analysis

All graphics were made, and all statistical analysis was done using Graph Pad Prism. Unless otherwise noted, bar graphs represent the mean ± standard error of the mean (SEM). For comparing statistical differences between means of multiple samples a standard two-way ANOVA was used. For comparisons between paired groups, a standard two-way ANOVA was used. For all comparisons, p-values were corrected for multiple comparisons using Tukey’s multiple comparisons test. Correlations were done using a Spearman correlation. Statistical tests used are noted in figure legends.

## 3. Results

### 3.1. Tumor EVs Program Macrophages to a Pro-Metastatic Phenotype In Vitro and In Vivo

**Figure 1:**
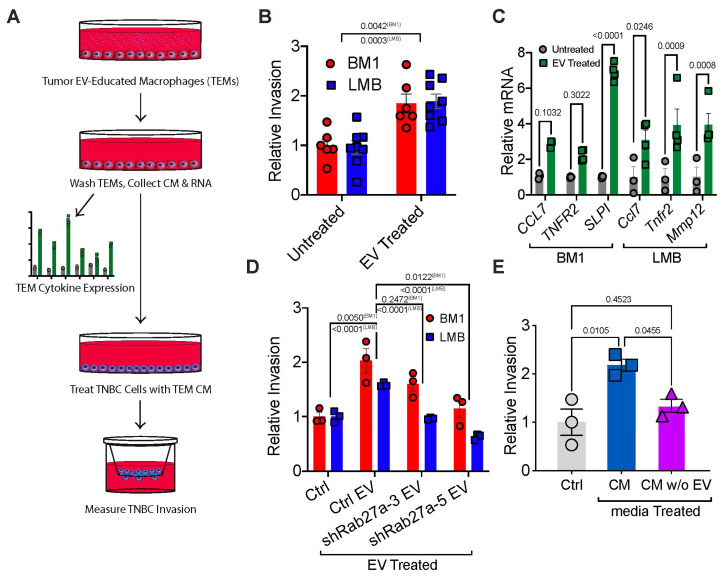
Tumor EVs are required to program macrophages to a pro-metastatic phenotype in vitro (**A**) Schematic describing the method by which TEMs were programmed with tumor EVs, followed by analysis of TEM gene expression and effect of TEMs to TNBC invasion. TNBC Cells BM1 and LMB were used for all experiments. P-values were calculated using a two-way *t*-test (**B**) Relative invasion of TNBC cells pre-incubated for 24 h with TEM conditioned media (*N* = 6). (**C**) qRT-PCR of *Ccl7*, *Tnfr2*, *Slpi*, and *Mmp12* in TEMs programmed with TNBC EVs compared to M-CSF alone (Control) with *Gapdh* as a loading control. P-values were calculated using a two-way ANOVA (*N* = 3 for LMB; *N* = 4 for BM1). (**D**) Relative invasion of TNBC cells pre-incubated for 24 h with TEM conditioned media (*N* = 3). TEMs were programmed with M-CSF (Control) or EVs from control (Ctrl EV) or sh-Rab27a tumor cells (shRab27a clones: sh3, sh5). P-values were calculated using a one-way ANOVA comparing changes in BM1 and LMB cells separately between groups (**E**) Relative BM1 invasion (mean +/− SEM) when cells were treated with TEM media programmed by an M-CSF control (control, grey), BM1 media with EVs (CM, red), or BM1 media with EVs removed (CM w/o EV, purple). *p*-values were calculated using a one-way ANOVA comparing both changes in both BM1 and LMB cells together between groups.

To investigate whether tumor EVs educate macrophages toward a pro-metastatic TAM phenotype, we treated bone marrow-derived macrophages (BMDMs) with tumor EVs for 48 h in the presence of macrophage colony stimulating factor (M-CSF), creating tumor EV-educated macrophages (TEMs) (Figure 1A, Figures S1 and S2; Appendix A). Isolated EVs were characterized per MISEV2018 guidelines (Figure S2A,B) [39]. Macrophages treated with tumor EVs drove tumor cell invasion at a higher rate than the untreated control (Figure 1B). Tumor EVs induced expression in macrophages of pro-invasive markers *Ccl7*, *Tnfr2*, *Mmp12*, and *Slpi* compared to an M-CSF only control (Figure 1C) [11].

To determine whether tumor EV secretion plays an active role in macrophage-induced tumor cell invasion, we knocked down *Rab27a*, a known regulator of the EV secretion machinery (Figure S3A) [22,26]. Expression of shRNAs for Rab27a in human BM1 and mouse LMB tumor cells decreased EV secretion by ~50% (measured by NTA), similar to previous reports [22,40] (Figure S3B–I). Considering that the Rab family of proteins are also regulators of endosomal sorting and membrane fusion, we also investigated if knockdown of Rab27a might affect other relevant cell properties. However, we observed no change in tumor cell growth, migration, or invasion of shRab27a cells relative to controls (Figure S4). Next, we educated macrophages using EVs from an equal number of control or Rab27a-knockdown tumor cells to generate TEMs, allowing differences in EV secretion to affect macrophage education. The TEMs from these two conditions were then used in our invasion assay (Figure 1A). We observed significantly reduced tumor cell invasion when TEMs were educated using EVs from either shRab27a BM1 and shRab27a LMB cells compared to controls (Figure 1D). Consistent with this result, tumor media depleted of EVs by ultracentrifugation was unable to program macrophages capable of driving tumor cell invasion (Figure 1E). Together, these results suggest that tumor EVs are a necessary component for programming macrophages to promote a pro-metastatic phenotype in vitro.

**Figure 2:**
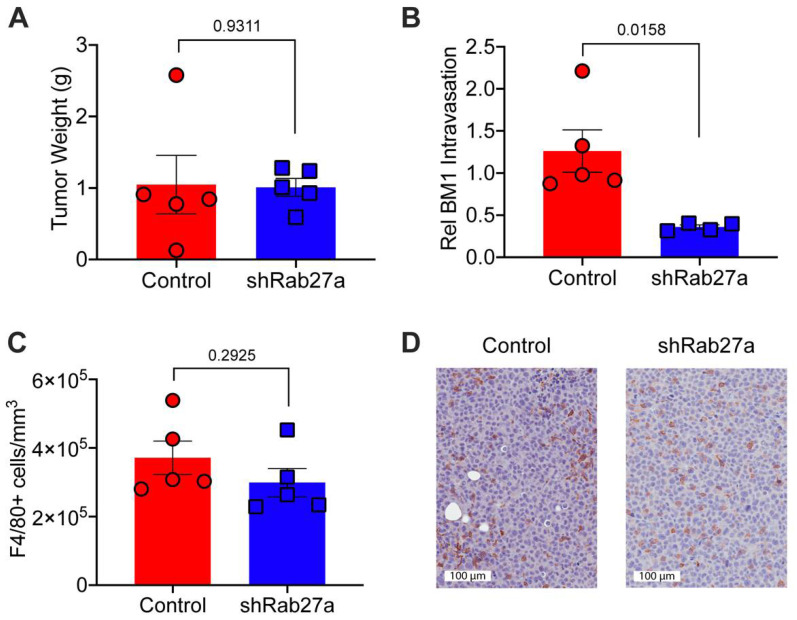
Tumor EVs generate TAMs that promote tumor intravasation in vivo (**A–D**) BM1+Control vector (Control, Red) or BM1+shRab27a (sh-5) (shRab27a, Blue) cells (2 × 10^6^) were injected into athymic nude mice. (**A**) Final tumor weights at sacrifice (*N* = 5). (**B**) Relative intravasation of tumor cells into the blood stream measured by quantifying the ratio of human *GAPDH* (tumor) to mouse *Gapdh* (white blood cells) by qRT-PCR (*N* = 5) from blood taken immediately prior to sacrifice. (**C**) Macrophage infiltration was quantified as the number of positive cells normalized to area using Aperio. (*N* = 5 per group). (**D**) Representative images of F4/80-stained tumor sections in Control and shRab27a tumors. P-values between two groups were calculated using a *t*-test.

To determine if reducing tumor EV secretion impacts in vivo macrophage polarization and metastasis, we injected BM1 tumor cells (+/− shRab27a) into the inguinal fat pads of athymic nude mice (with an intact innate immune system [41,42]). We utilized shRab27a (clone sh-5) as it produced the most robust change in EV secretion and in vitro macrophage programming. After tumors were established in the mice, we measured tumor size, tumor cells circulating in the blood stream, and macrophages recruited to tumors. Reducing EV secretion through Rab27a knockdown did not affect the size of BM1 tumors (Figure 2A). However, fewer tumor cells were found in circulation in the shRab27a group compared to control (Figure 2B). This is consistent with a role of tumor EVs in the early stages of metastasis (tumor cell intravasation into the bloodstream). As previously noted, shRab27a did not affect cell autonomous invasion nor migration (Figure S4B,C), suggesting that the effects on tumor intravasation are due to changes in cells within the tumor microenvironment, not the inherent invasive ability of the tumor cells. We therefore wanted to explore whether reducing tumor EV secretion affected the recruitment of TAMs. When we stained for F4/80, a surface glycoprotein frequently found on mouse macrophages, we found no significant difference in the number of TAMs recruited to control versus shRab27a tumors (Figure 2C,D).

**Figure 3:**
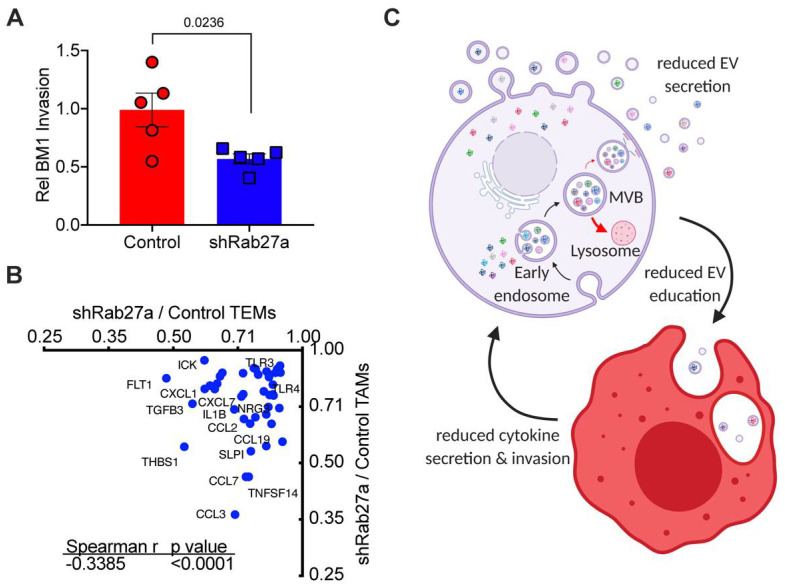
Tumor EVs regulate macrophage phenotype in vivo (**A**) Invasion assay of BM1 cells treated with CM of TAMs derived from control or shRab27a expressing BM1 tumors (*N* = 5). (*p*-values were calculated using a one-way ANOVA). (**B**) Spearman correlations of macrophages proteins assayed using an L-308 Cytokine Array (*N* = 3 mice (TEMs) or 5 mice (TAMs), pooled for array analysis). Zoom-in of the lower left quadrant of a Spearman correlation of cytokines regulate by shRab27a EV secretion in TEMs vs TAMs (showing the 42 proteins reduced by shRab27a in both TEMs and TAMs). *X*-axis: comparison of proteins in shRab27a to Control TEMs. *Y*-axis: comparison of proteins in shRAb27a versus Control TAMs. Correlation determined using a Spearman test. (**C**) Schematic summarizing Rab27a-dependent EV secretion and subsequently macrophage programming. Resulting EV programmed macrophages drive increase tumor cell invasion. Created using Biorender.com (accessed on 2 June 2021).

Because we saw no differences in macrophage number recruited to tumors when tumor Rab27a expression was reduced, we next sought to determine how EV education of macrophages alters their phenotype in vivo. We isolated macrophages from control and shRab27a BM1 tumors (Figure 2D). We then collected conditioned media from the isolated macrophages and tested the ability of secreted factors in the media from these macrophages to drive tumor cell invasion. We found that macrophages from shRab27a tumors failed to drive tumor cell invasion at the same rate as macrophages from control tumors (Figure 3A). Because this was the same phenotype observed in the in vitro programmed macrophages, we decided to compare how shRab27a altered macrophage biology in vitro to in vivo. To test this, we compared differential expression of cytokines in TEMs (in vitro) to TAMs (in vivo) upon reduction of EV secretion from tumor cells by shRab27a. We observed that ~30% of differentially expressed proteins overlapped between our in vitro TEM and in vivo TAM polarization experiments. These included CCL7, CCL19, CXCL7, NRG3, OPN, HGF, and TGFB3 (Figure 3B, Figure S5A–C), known drivers of immune evasion and metastasis [11,43,44]. These findings provide further support that tumor EVs are an essential component for programming macrophages toward a pro-metastatic TAM phenotype in the tumor microenvironment. Thus, reducing tumor EV secretion sufficiently decreases both macrophage cytokine secretion and macrophage-driven tumor cell invasion (Figure 3C).

### 3.2. Tumor CCL5 Expression Regulates EV Secretion and EV Education of Macrophages

**Figure 4:**
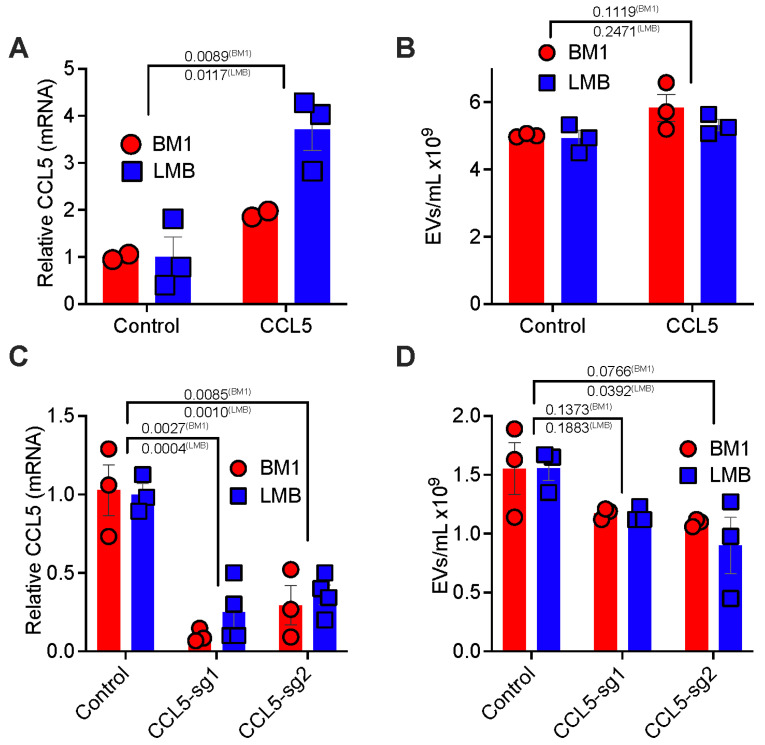
Tumor CCL5 expression regulates EV secretion (**A**) qRT-PCR of CCL5 in BM1 using GAPDH as loading control or ddPCR of Ccl5 in LMB cells. Relative expression of *CCL5*/*Ccl5* in CCL5 over-expressing cells (CCL5) is compared to those with a control vector (Control). *p*-value was determined using a two-tailed *t*-test (*N* = 3). (**B**) Number of EVs/mL determined by NTA analysis using a Nanosight in BM1 and LMB cells either expressing a control vector (control) or *CCL5* over-expressing (CCL5). *p*-value was determined using a two-tailed *t*-test (*N* = 3). (**C**) qRT-PCR of *CCL5* in BM1 using *GAPDH* as loading control or ddPCR of *Ccl5* in LMB cells. Relative expression of *CCL5*/*Ccl5* in BM1 and LMB cells using CRISPR/Cas9 KO (BM1) or CRISPR/dCas9-KRAB (LMB). *CCL5* reduced cells (sg-1, sg-2) are compared to those with a control vector (Control). *p*-value was determined using a one-way ANOVA (*N* = 3). (**D**) Number of EVs/mL determined by NTA analysis using a Nanosight in BM1 and LMB cells either expressing a control vector (control) or CRISPR/Cas9 KO (BM1), CRISPR/dCas9-KRAB (LMB) with a *CCL5*/*Ccl5* targeting guide RNA (sg-1, sg-2). *p*-values were determined using a one-way ANOVA (*N* = 3).

To explore the tumor intrinsic mechanism responsible for tumor EV-driven macrophage education, we investigated whether tumor cytokines that recruit macrophages also participate in macrophage education to promote metastasis. We focused on the potential role of CCL5, a chemokine that we and others previously identified to be a key mediator of macrophage recruitment in TNBC [10,11]. First, we tested if direct CCL5 stimulation of macrophages was sufficient to drive them toward a pro-tumor phenotype. Indeed, neither M-CSF nor M-CSF+CCL5 stimulation of macrophages promoted tumor cell invasion **(**Figure S6A). Additionally, neither treatment induced expression of pro-metastatic markers *Ccl7*, *Mmp12*, and *Grn* (Figure S6B). However, direct stimulation of tumor cells with CCL5 led to an increased macrophages capable of driving tumor cell invasion (Figure S6C). We hypothesized that tumor *CCL5* expression regulates macrophage reprogramming through an indirect mechanism. We first tested how increased tumor *CCL5* expression regulated tumor EV secretion. When *CCL5* was overexpressed (*CCL5^high^*) in human BM1 or mouse LMB TNBC cells (Figure 4A), we observed a slight but not significant increase in EV secretion from comparable tumor cell numbers (Figure 4B and Figure S6). Conversely, reduction of *CCL5* expression through either CRISPR-Cas9 (BM1 cells) or CRISPR-dCas9-KRAB (LMB cells) (Figure 4C) decreased tumor EV secretion with one guide RNA (Figure 4D and Figure S7). When we examined EVs from CRISPR-dCas9-KRAB KD cells, we found a significant reduction in *Ccl5* copies present (Figure S7I).

**Figure 5:**
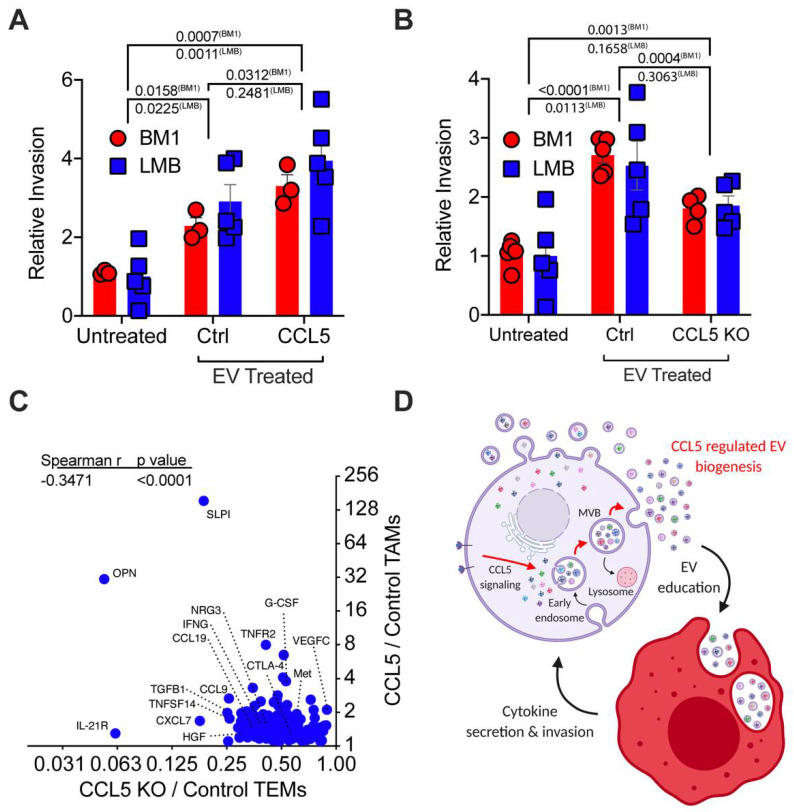
Tumor CCL5 expression regulates EV programming of macrophages. (**A**) Relative invasion of BM1 and LMB cells treated with TEM conditioned media (CM). TEMs were programmed with CSF-1 alone (Untreated), EVs from tumor cells with control vector (Ctrl), or EVs from tumor cells over-expressing *CCL5* (CCL5). *p*-value was obtained using a one-way ANOVA comparing changes in invasion in each cell line upon TEM CM stimulation (*N* = 3 BM1, *N* = 5 LMB). (**B**) Relative invasion of BM1 and LMB cells treated with TEM conditioned media (CM). TEMs were programmed with CSF-1 alone (Untreated), EVs from tumor cells with control vector (Ctrl), or EVs from tumor cells with reduced *CCL5* through CRISPR (CCL5 KO). *p*-value was obtained using a one-way ANOVA comparing changes in invasion in each cell line upon TEM CM stimulation (*N* = 5). (**C**) Spearman correlations of macrophages proteins assayed using an L-308 Cytokine Array (*N* = 3 mice (TEMs) or 5 mice (TAMs), pooled for array analysis). Zoom-in of the upper left quadrant of a Spearman correlation of cytokines regulated by *CCL5* in TEMs vs TAMs. Graph shows relative expression of proteins in BM1 *CCL5* KO TEMs to BM1 Control TEMs. *Y*-axis: relative expression of proteins in BM1+CCL5 TAMs compared to BM1 Control TAMs. *CCL5* TAM data set was used from previous publication for this analysis [11]. (**D**) Schematic summarizing *CCL5* regulation of EV secretion and subsequently macrophage programming. Resulting *CCL5*-EV programmed macrophages drive increase tumor cell invasion. Created using Biorender.com (accessed on 2 June 2021).

Knowing that *CCL5* regulates the number of EVs that tumor cells secrete, we also wanted to test if it regulates the cargo of the EVs and subsequently their impact on macrophages. When we treated macrophages with the same number of control and *CCL5^high^* tumor EVs (quantitated by NTA), there was an approximately 50% increase in TEM-driven tumor cell invasion (Figure 5A). These data clearly indicate that the cargo in EVs, and not the number of EVs, drive functional programming of macrophage to drive tumor cell invasion. Conversely, reduction of *CCL5* expression through either CRISPR-Cas9 (BM1 cells) or CRISPR-dCas9-KRAB (LMB cells) (Figure 4C) decreased TEM-mediated tumor cell invasion (Figure 5B). To determine the effect of *CCL5* on macrophage reprogramming, we analyzed cytokines in the conditioned media from TEM or TAM using an L308 mouse cytokine array (Figure S8). We generated TEMs in vitro using EVs from sg*CCL5* or control TNBC cells, and identified TEM cytokines whose expression was decreased upon loss of tumor cell *CCL5* expression (Figure S8A). We then compared them to cytokines in TAMs from *CCL5^high^* in vivo tumors [11] (Figure 5C and Figure S8B). Interestingly, CCL7, CCL19, CXCL7, NRG3, SLPI, OPN, and HGF in macrophages are upregulated in response to increased tumor *CCL5* expression (Figure 5C). Overall, these experiments suggest that tumor *CCL5* not only enhances the number of EVs but also alters the cargo of EVs. Analysis of these differentially expressed cytokines indicated that pro-metastatic osteopontin (OPN) [44] and SLPI [45,46,47,48] in both TEMs (in vitro) and TAMs (in vivo) are most strongly induced by tumor *CCL5*. Our previous work had established *OPN* and *SLPI* as markers of macrophages activated by *CCL5* [11], and both of these factors have been shown to directly regulate tumor cell invasiveness [44,45,46,47,48]. These data represent a positive feedback loop between tumor and macrophage mediated by CCL5 and EVs (Figure 5D).

### 3.3. Metastatic Tumor EVs Educate Macrophages to Promote Tumor Metastasis In Vivo

**Figure 6:**
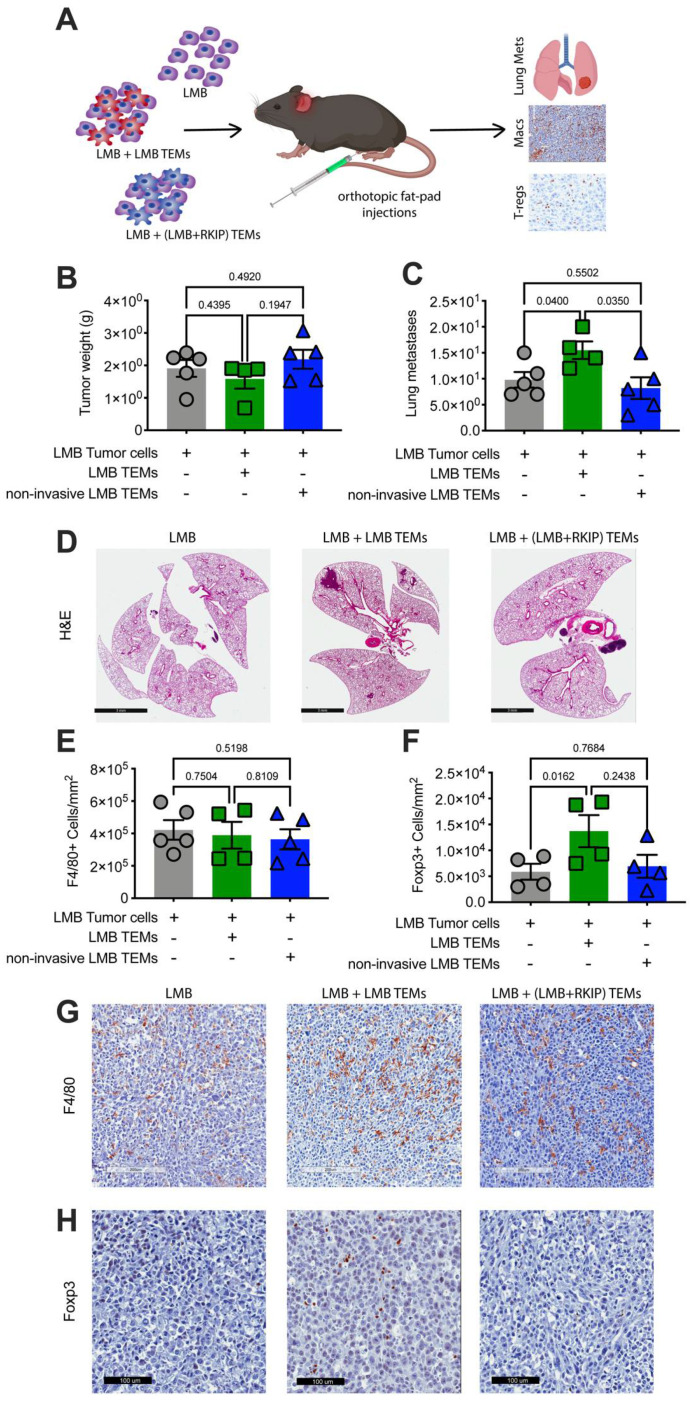
Tumor EVs promote metastasis through macrophages. (**A**) Either 0.5 × 10^6^ LMB EV programmed or LMB (overexpressing RKIP) EV programmed TEMs were co-injected with tumor cells as indicated. Final tumor weights (**B**) and number of metastases (**C**) per mouse are shown for LMB tumors alone (grey, *N* = 5), LMB tumor cells with LMB programmed TEMs (green, *N* = 4), and LMB tumor cells with LMB (RKIP overexpressing, non-metastatic) programmed TEMs (blue, *N* = 5). (**D**) Representative images of lung metastases from each group. Final number of macrophages (**E**) and Foxp3+ T-regs (**F**) per mouse are shown for LMB tumors alone (grey, *N* = 5), LMB tumor cells with LMB programmed TEMs (green, *N* = 4), and LMB tumor cells with LMB (RKIP overexpressing, non-metastatic) programmed TEMs (blue, *N* =5). Representative images of F4/80+ macrophages (**G**) and Foxp3+ T-regs (**H**) from each group. (All *p*-values were calculated utilizing a *t*-test).

To assess whether in vitro-generated TEMs are sufficient to promote metastasis in a syngeneic mouse model, we co-injected mouse TNBC cells (LMB) and LMB EV-educated macrophages (TEMs) into the inguinal fat pad of C57Bl/6 mice (Figure 6A). Co-injected TEMs had no effect on LMB tumor size (Figure 6B) but did increase both number and size of lung metastases compared to LMB tumor cells injected alone (Figure 6C,D). We observed a similar enhancement of the metastatic phenotype when we co-injected human BM1 tumor cells with in vivo-generated TAMs isolated from *CCL5*^high^ BM1 tumors (Figure S9).

To determine whether the metastatic state of the tumor cells influences the ability of the educated macrophages to promote metastasis, we evaluated non-metastatic cells as a source of EVs (Figure S10, Appendix B). To generate a non-metastatic cell line for TNBC, we first expressed the metastasis suppressor, RKIP, [11] in LMB cells (Figure S10, Appendix B). We then used these RKIP-expressing LMB cells to produce EVs to program macrophages in vitro. In this case, co-injection of LMB cells with TEMs generated by these non-invasive, non-metastatic tumor EVs (RKIP+ LMB EVs) showed no significant change in lung metastases or tumor size compared to the TEM (−) control (Figure 6). These results suggest that in vitro EV-programmed TEMs phenocopy the metastatic state of the parental tumor cells from which the EVs are derived.

Although co-injection of TNBC tumor cells with in vitro-generated TEMs altered the function of tumor associated macrophages, recruitment of macrophages to the tumor did not change. When we stained for the macrophage marker F4/80, we found that the total number of macrophages in the tumor were the same between control tumors and those co-injected with TEMs (Figure 6E,G). This result suggests that the phenotype of the EV-educated macrophages, not simply the presence of macrophages alone, is driving increased metastasis.

Because EV programming of macrophages resulted in the secretion of cytokines known to recruit T-regs (CXCL1, CCL5, CCL19), we also immunostained tumors for the T-reg marker Foxp3. Co-injection of metastatic tumor cells (LMB) with TEMs generated by metastatic tumor EVs significantly increased the number of Foxp3+ T-regs compared to control (Figure 6F,H). Conversely, co-injection of metastatic tumor cells (LMB) with TEMs generated using non-metastatic tumor EVs (LMB+RKIP EVs) showed no change in T-reg recruitment (Figure 6F,H). This result indicates that EVs, as a key regulator of macrophage phenotype, also indirectly affect the tumor microenvironment in vivo, driving it toward an immunosuppressive phenotype by altering macrophage biology to recruit T-regs.

### 3.4. Clinical Significance of Tumor EV Macrophage Programming

**Figure 7:**
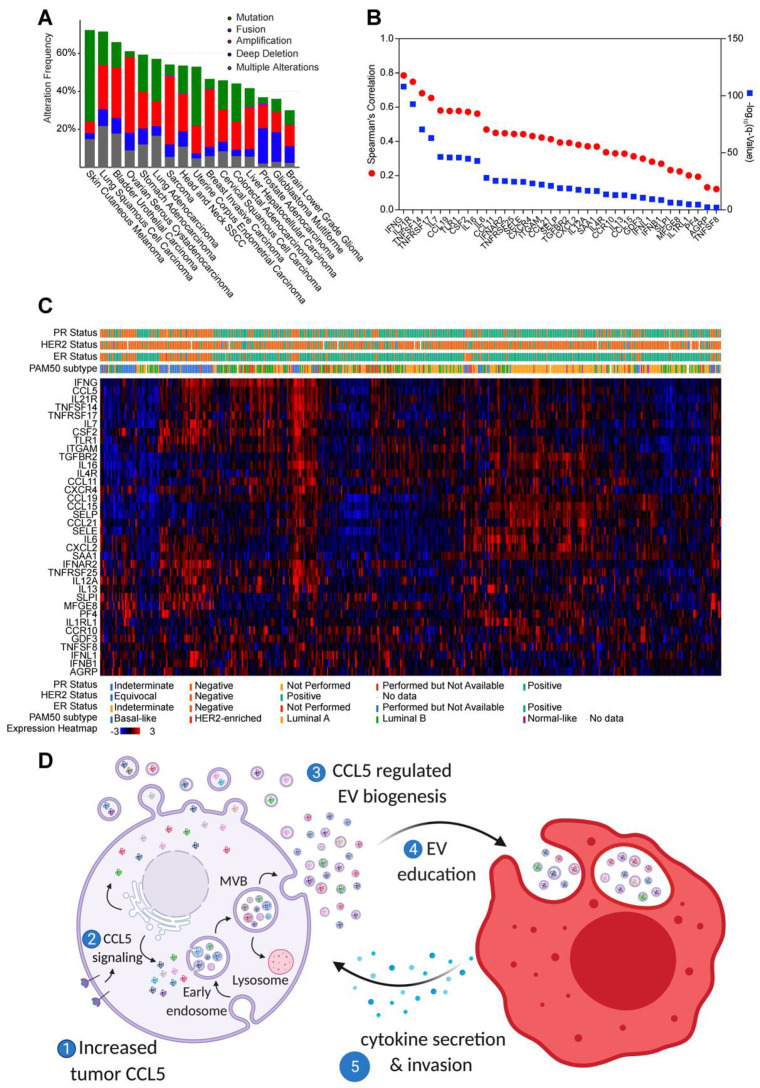
Clinical Significance of EVs as Biomarkers. (**A**) Top 16 tumor types with genetic alterations in genes we found differentially expressed in macrophages by tumor *Rab27a* and *CCL5*. (**B**) Spearman correlation of genes regulated in macrophages by tumor *Rab27a* and *CCL5* to *CCL5* in all TCGA breast samples (*N* = 1100) (**C**) Expression heatmap of TCGA breast cancer patients for mRNAs induced in macrophages by tumor *Rab27a* and *CCL5*. Patients were clustered by co-expression in cBioPortal, and ER/PR status by IHC is shown. (**D**) Schematic depicting tumor CCL5 expression regulating EV stimulation of macrophages leading to secretion of CCL7, CSF2, IL-21R, IL7, CXCR4, CCL19, IFNG, and OPN, increasing tumor metastasis.

To evaluate the clinical significance of tumor EV programming of macrophages, we looked in our data for cytokines commonly regulated by tumor EV secretion and tumor *CCL5* expression (Table S1; Figure 3B and Figure 5C). DAVID analysis [49] of this gene set indicated enrichment in macrophage activation, chemotaxis, cell proliferation, negative regulation of apoptosis, and cell signaling (Figure S11). These genes in this set regulated both by EV numbers and tumor CCL5 expression are altered in a variety of solid tumor types, including melanoma, lung, bladder, ovarian, stomach, head and neck, uterine, breast, cervical, colorectal, liver, and prostate cancer as well as high- and low-grade gliomas (Figure 7A). Analysis of the expression of CCL5-regulated cytokines in patient breast tumors revealed ~75 percent were highly correlated with *CCL5* expression in TCGA (Figure 7B). When we examined the expression of these CCL5-regulated cytokines across breast tumors, we found that high expression of cytokines and chemokines clustered with high expression of *CCL5* in triple-negative/basal-like breast tumors (Figure 7C and Figure S12). Together, these results suggest that increased *CCL5* expression in tumors leads to increased and altered EVs from tumor cells. These EVs then educate macrophages, which then secrete cytokines and other pro-metastatic factors that alter the tumor microenvironment and drive tumor metastasis (see summary schematic in Figure 7D).

## 4. Discussion

Here we demonstrate that tumor EVs are an integral component for education of macrophages capable of driving metastasis in TNBC. *CCL5* regulates both the secretion of EVs from tumor cells, as well as their ability to program macrophages. Analyses of clinical breast cancer data showed significant correlation between expression of *CCL5* and a set of genes correspondingly regulated in macrophages by tumor EVs and *CCL5* expression. These genes were specifically enriched in TNBC or basal-like patients. The metastatic state of the tumor was translated to macrophages through EV programming, suggesting that the tumor EVs are capturing and transmitting the metastatic state of the tumor to the microenvironment.

This work also shows that *CCL5* regulates not only macrophage recruitment but also programming. Studies by our group and others have demonstrated that *CCL5* plays a critical role in the recruitment of TAMs that drive tumor metastasis [10,11] as well as directly stimulating tumor progression [16]. By contrast, the present work shows that CCL5 protein secreted from tumors does not have a direct effect on macrophage programming to a TAM phenotype. Instead, these data suggest that *CCL5* autocrine signaling promotes generation of tumor EVs that reprogram macrophages to a more metastatic, TAM function. Changing *CCL5* expression in tumors alters cargo such as *CCL5* mRNA in EVs as well as the number of EVs secreted. Additionally, our study shows that is not the number of macrophages recruited to the tumor, but their phenotype that is important for tumor metastasis. These findings further demonstrate that *CCL5* plays a novel role, through EVs, in altering macrophage function rather than just recruitment. Macrophages altered by tumor *CCL5* expression and EVs have increased expression of several genes known to drive invasion and metastasis, including *OPN* [44], *SLPI* [45,46,47,48], *HGF* [43], and *NRG3* [50]. There are several possible reasons why activation by CCL5-stimulated EVs rather than direct CCL5 interaction is required to educate tumor-recruited macrophages. For example, even in the context of a single macrophage receptor, EVs can express multiple ligands and, therefore, may bind with higher affinity.

Several of the proteins that are regulated in TEMs and TAMs by tumor EVs and *CCL5* are potential chemotactic factors for other cell types within the microenvironment such as T-regs. In particular, *CCL19* is a chemotactic factor involved in recruiting CD4+CD25+CD69- T-regs to zones of T cell infiltration in the tumor micro-environment [51,52,53]. Expression of CXCL1 also recruits T-regs to the tumor microenvironment through CXCR2 signaling [54]. In addition, interferon-γ (*IFNG*) has been implicated in T-reg polarization [55,56,57,58]. Since *CCL19* and *IFNG* are highly correlated with *CCL5* expression in patient breast tumors, these may be most relevant to T-reg infiltration of tumors in response to *CCL5*. Our data suggest that tumor EVs not only impact TAM phenotypes directly, but also alter their ability to attract and interact with other cells that contribute to evasion of immune detection.

These results highlight the importance of *CCL5* and tumor EVs as key potential targets for tumor immunotherapy. Previous work has established that tumors infiltrated with T cells, T-regs, and macrophage recruitment may respond well to checkpoint blockade therapy [59,60]. The genes we see regulated in macrophages by *CCL5* altered EVs are highly correlated with expression of *CD8*, *CD4*, *CD25*, *LAG3*, *Foxp3*, and *IFNG*, as well as macrophage markers, in TNBC patients. This suggests that tumor cells EV protein or RNA markers may also be prognostic for response to checkpoint blockade therapies. Because of the strong evidence of a biological role these EVs play in altering the immune microenvironment, it furthers their potential for use as biomarkers of tumor outcome as well as potential response to immunotherapy.

Overall, our results show that tumor-associated macrophages, which play a key role in enabling tumor cells to invade surrounding tissue, intravasate into vessels, and metastasize, rely on tumor EVs from metastatic cells to be programmed toward a pro-metastatic phenotype. As such, EVs can play an even more pivotal role in metastatic progression than previously realized. Targeting tumor EVs will not only be essential to reduce the formation of the pre-metastatic niche, but also block the education of the primary tumor microenvironment including macrophages to reduce tumor burden and tumor cell dissemination.

## Data Availability

Data generated or analyzed during this study are included in this published article (and its supplementary information files). Reasonable requests for other raw data supporting this work can be made to Dr. Rosner.

## Supplementary Materials

The following are available online at https://www.mdpi.com/article/10.3390/cancers13143459/s1, Figure S1: Schematic describing our experimental design for in vitro EV analysis; Figure S2: Validation of small-EV markers by flow, immunoblot, and scanning EM; Figure S3: Reduction in EV secretion in BM1 and LMB cells upon Rab27a Knock-Down; Figure S4: Rab27a Knock-Down in BM1 cells does not alter tumor growth, migration, or invasion; Figure S5: Cytokine array analysis of EV-programmed macrophages produced from control and shRab27a tumor cells; Figure S6: CCL5 protein alone not sufficient to drive macrophage programming; Figure S7: CCL5 regulates EV secretion in BM1 and LMB tumor cells; Figure S8: Cytokine array analysis of macrophages programmed with either control or CCL5-Knock-Down EVs; Figure S9: CCL5 recruited TAMs increase both tumor cell growth and metastasis; Figure S10: Tumor RKIP expression does not alter EV secretion, but blocks pro-tumor programming of macrophages by tumor EVs; Figure S11: DAVID analysis macrophage genes regulated by EV programming and tumor CCL5 expression; Figure S12: Heatmap of z-score normalized gene expression in METABRICK individual patient breast cancer samples.

## Appendix A

BMDMs treated with 20 ng/mL of M-CSF served as a negative control (Figure S1). For these studies, we used the TNBC cell line BM1, a highly invasive bone-metastatic variant of MDA-MB-231 [36], or mouse TNBC cells, E0771-LMB (referred to as LMB) [37]. While there are many human lines for TNBC, we utilized BM1 cells because they are highly invasive and because their signaling pathway has been extensively studied by our lab. We previously showed that RKIP regulates CXCR4 and OPN through HMGA1 and BACH1 through the miRNA let-7 [61]. Additionally, in our previous work, we showed that RKIP regulated macrophage recruitment by downregulating CCL5 expression in BM1 cells [11]. LMB cells were utilized because of their highly invasive nature, as well as similarity to human TNBC expression compared to other syngeneic models [37]. EVs were enriched from the TNBC cell line BM1, a highly invasive bone-metastatic variant of MDA-MB-231 [36], or mouse TNBC cells, E0771-LMB (referred to as LMB) [37] using iZon qEV columns (Figure S2A–C). To determine how to best derive EVs, we tested whether EVs isolated from tumor cells ex vivo compared to in vitro or in serum free vs exosome depleted FBS (Gibco) altered macrophage biology. We found no difference between macrophages treated with EVs from ex vivo or in vitro tumor cells (Figure S2D) or between EVs isolated in serum free culture media compared to grown with media supplemented with exosome depleted FBS (Figure S2E). We moved forward with isolation of EVs in serum free media from in vitro tumor cells. BMDMs were treated with 20 ng/mL M-CSF with 1 × 10^8^ EVs/mL from BM1 tumor cells or 1 × 10^9^ EVs/mL for LMB cells for 48 h to generate tumor EV-educated macrophages (TEMs). Once TEMs were educated, we used two assays to characterize TEMs. (1) RNA or secreted factors were isolated from TEMs and assayed for cytokine expression of pro-metastatic macrophage markers (*Ccl7, Tnfr2,* or *Mmp12*) [11,62,63]. (2) TEMs were washed and incubated in serum free media to assess the effect of TEM secreted factors in our invasion assay.

## Appendix B

To block the metastatic ability of tumor cells, we overexpressed the metastasis suppressor gene Raf kinase inhibitor protein (RKIP, PEBP1). We have extensively demonstrated the ability of RKIP to block both early and late metastasis [38], regulate HMGA2 and BACH1 signaling [61,64], as well as regulate macrophage recruitment to TNBC through regulation of tumor CCL5 expression [11]. When we overexpressed RKIP in BM1 and LMB cells, we found no significant change in the number of EVs secreted from either cell line (Figure S10A–G). However, TEMs programmed from RKIP over-expressing cells were not able to significantly drive tumor cell invasion, while TEMs created with EVs from control cells were able to significantly drive invasion (Figure S10G–I). When we examined expression of pro-metastatic macrophage marker genes we previously established [11], we found that TEMs created with EVs from RKIP-overexpressing cells had significantly lower expression of *Grn*, *Mmp12*, and *Ccl7* compared to TEMs created with EVs from control tumor cells (Figure S10I). Additionally, TEMs created with EVs from RKIP-overexpressing cells had similar levels of expression of *Grn*, *Mmp12*, and *Ccl7* compared to TEMs created with EVs from the normal mammary epithelial cell line 184A1 (Figure S10I).
