# Supplementary material for: Tumor Extracellular Vesicles Regulate Macrophage-Driven Metastasis through CCL5
Source: Cancers (Basel). 2021 Jul 10;13(14):3459. doi: 10.3390/cancers13143459 (PMC8303898; doi:10.3390/cancers13143459)
Supplement: Supplementary file 1 [file cancers-13-03459-s001.zip › Figure S1.pdf]

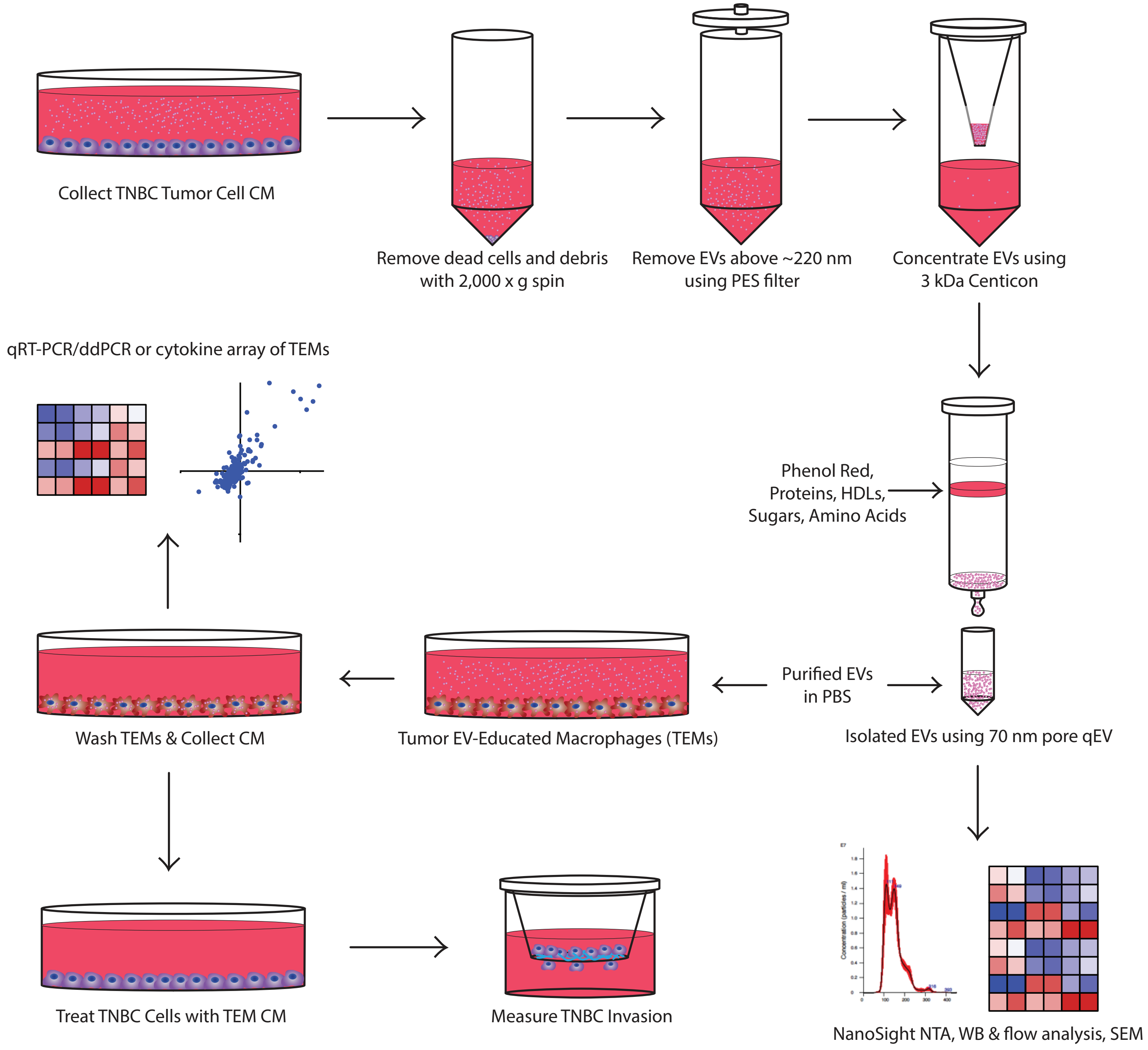

Figure S1: Schematic describing our experimental design for *in vitro* EV analysis:

- EV isolation method from TNBC CM using qEV columns
- EV analysis using NanoSight NTA as well immunoblot, flow, and SEM
- TEM programming with isolated EVs
- Gene expression analysis of TEMs using qRT-PCR/ddPCR or cytokine arrays
- Effect of TEM CM on tumor cell invasion
