# Supplementary material for: Tumor Extracellular Vesicles Regulate Macrophage-Driven Metastasis through CCL5
Source: Cancers (Basel). 2021 Jul 10;13(14):3459. doi: 10.3390/cancers13143459 (PMC8303898; doi:10.3390/cancers13143459)
Supplement: Supplementary file 1 [file cancers-13-03459-s001.zip › Figure S10.pdf]

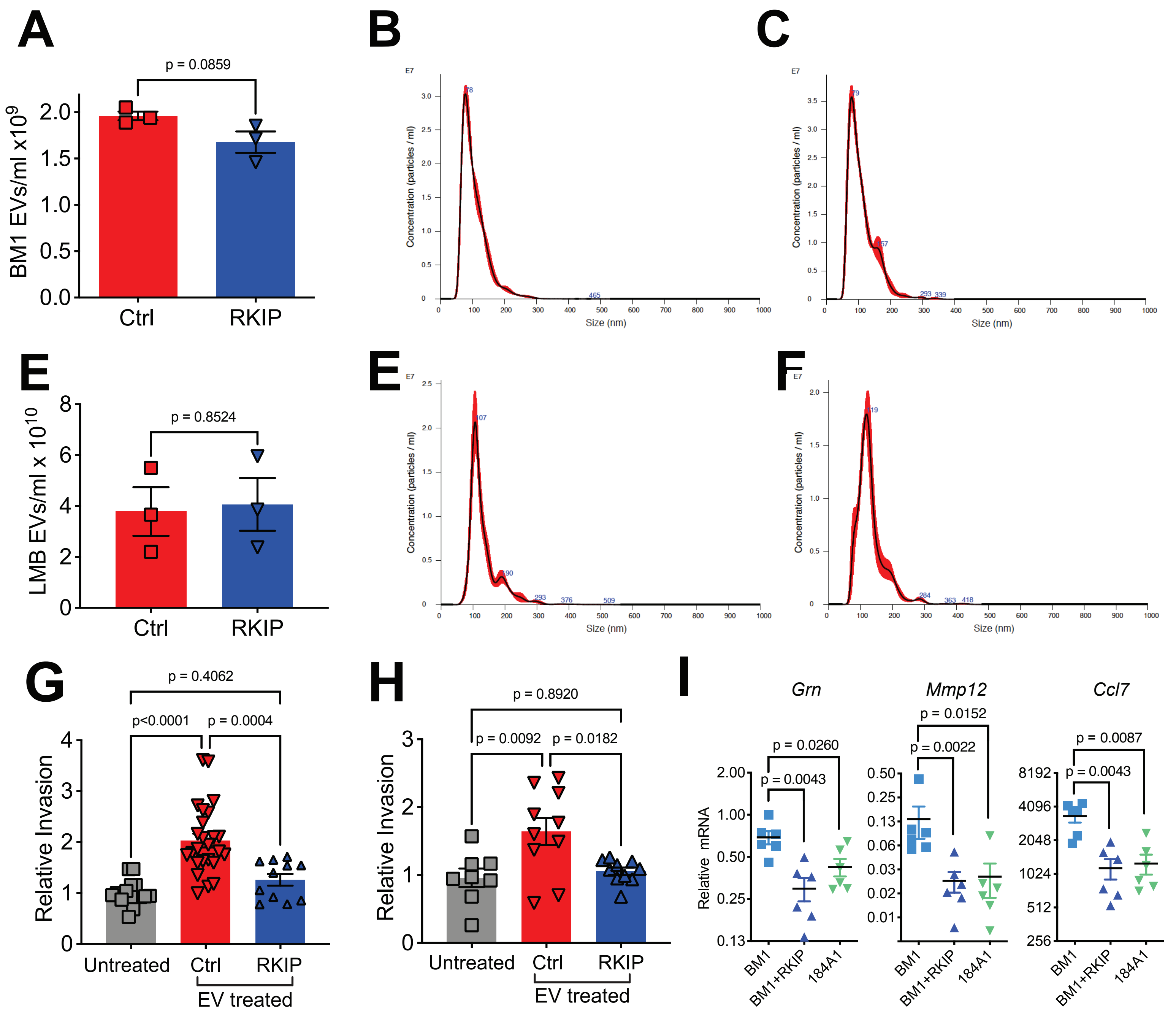

Figure S10: Tumor RKIP expression does not alter EV secretion, but blocks pro-tumor programming of macrophages by tumor EVs A) Mean +/- SEM of EV concentration in three independent replicates of BM1 cells expressing either control (pCDH1, black) or RKIP (blue) vectors. **(B-C)** Represenative nanosight graphs of EV concentrations and size in BM1 cell media after a 2,000 x g spin and 220 um filter for each cell type expressing either pCDH1 **(B)** or RKIP **(C)**. **D)** Mean +/- SEM of EV concentration in three independent replicates of LMB cells expressing either control (pCDH1, black) or RKIP (blue) vectors. **(E-F)** Represenative nanosight graphs of EV concentrations and size in LMB cell media after a 2,000 x g spin and 220 um filter for each cell type expressing either pCDH1 **(E)** or RKIP **(F)**. **G)** Relative invasion in BM1 cells treated with TEM conditioned media. TEMs programed with either M-CSF alone (untreated, grey), BM1 vector control EVs (Ctrl, red), or BM1+RKIP EVs (RKIP, blue). **H)** Relative invasion in LMB cells treated with TEM conditioned media. TEMs programed with either M-CSF alone (untreated, grey), LMB vector control EVs (Ctrl, red), or LMB+RKIP EVs (RKIP, blue). **I)** Relative mRNA values of *Grn*, *Mmp12*, and *Ccl7* normalized to *Gapdh* in TEMs programmed by tumor EVs, BM1 (light blue) or BM1+RKIP (dark blue), or normal mammary cell line 184A1 EVs (green). P-values calculated between groups
