# Supplementary material for: Tumor Extracellular Vesicles Regulate Macrophage-Driven Metastasis through CCL5
Source: Cancers (Basel). 2021 Jul 10;13(14):3459. doi: 10.3390/cancers13143459 (PMC8303898; doi:10.3390/cancers13143459)
Supplement: Supplementary file 1 [file cancers-13-03459-s001.zip › Figure S11.pdf]

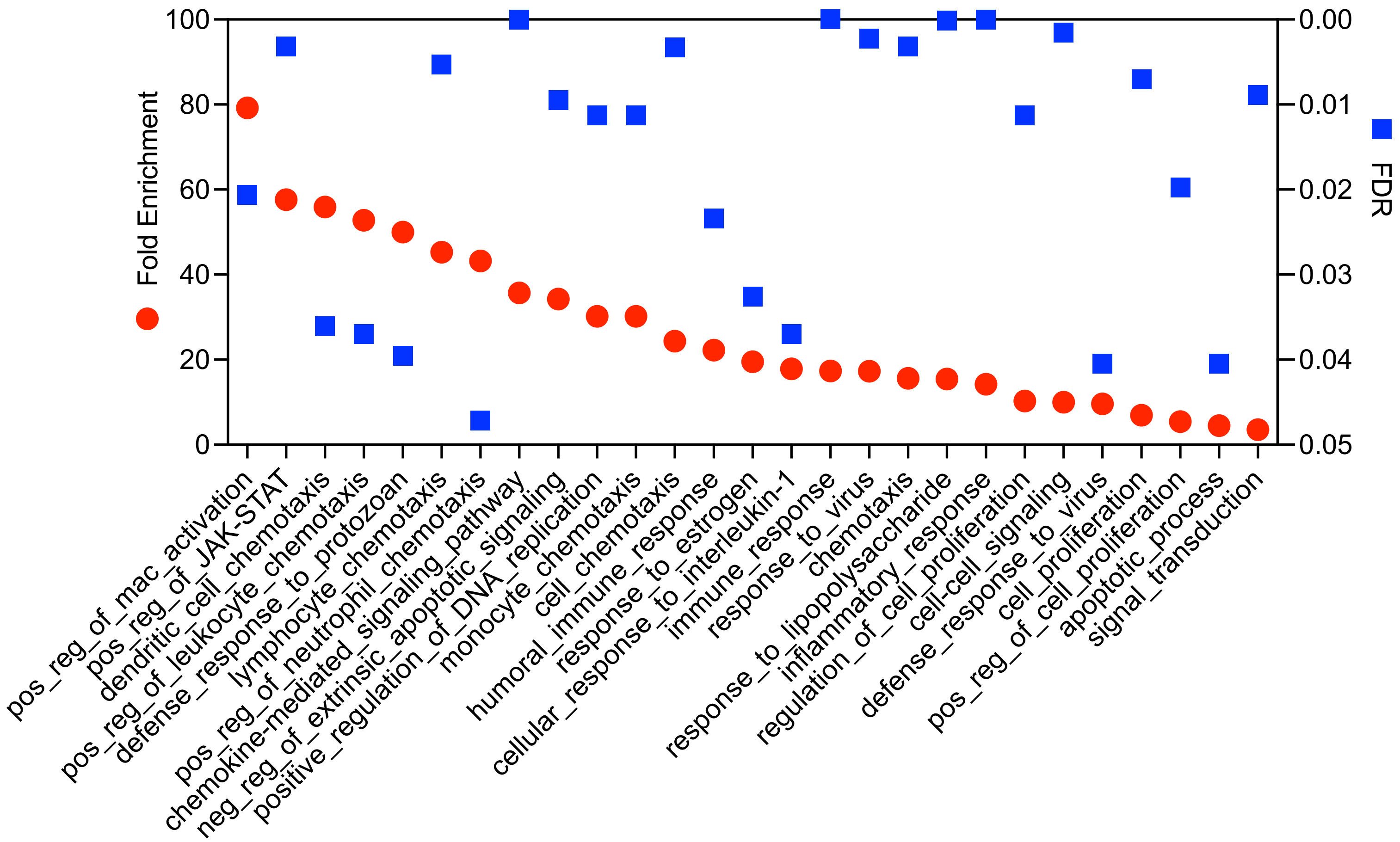

Figure S12: DAVID analysis macrophage genes regulated by EV programming and tumor CCL5 expression Shown in red are the number of genes out of the set of 50 enriched per category; shown in blue are the -log p-value of enrichment for each category. Only categories with greater than 10 genes are shown; the top 23 categories are shown.
