# Supplementary material for: Tumor Extracellular Vesicles Regulate Macrophage-Driven Metastasis through CCL5
Source: Cancers (Basel). 2021 Jul 10;13(14):3459. doi: 10.3390/cancers13143459 (PMC8303898; doi:10.3390/cancers13143459)
Supplement: Supplementary file 1 [file cancers-13-03459-s001.zip › Figure S12.pdf]

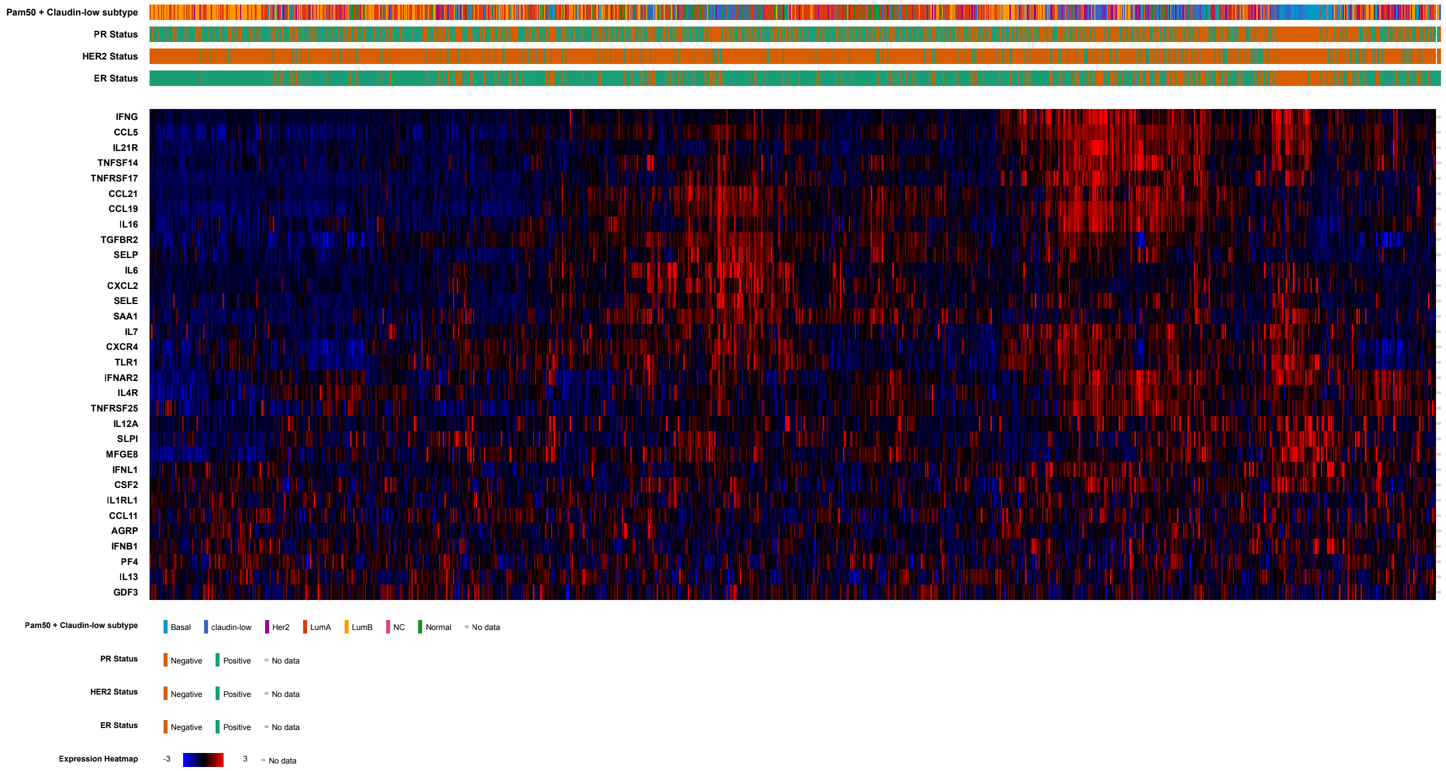

Figure S13: Heatmap of z-score normalized gene expression in METABRICK individual patient breast cancer samples Gene expression is shown in the metabrick dataset for the list of genes regulated by by EVs as well as tumor CCL5 expression. These genes cluster around high CCL5 expression after performing an unsupervised clustering.
