# Supplementary material for: Tumor Extracellular Vesicles Regulate Macrophage-Driven Metastasis through CCL5
Source: Cancers (Basel). 2021 Jul 10;13(14):3459. doi: 10.3390/cancers13143459 (PMC8303898; doi:10.3390/cancers13143459)
Supplement: Supplementary file 1 [file cancers-13-03459-s001.zip › Figure S2.pdf]

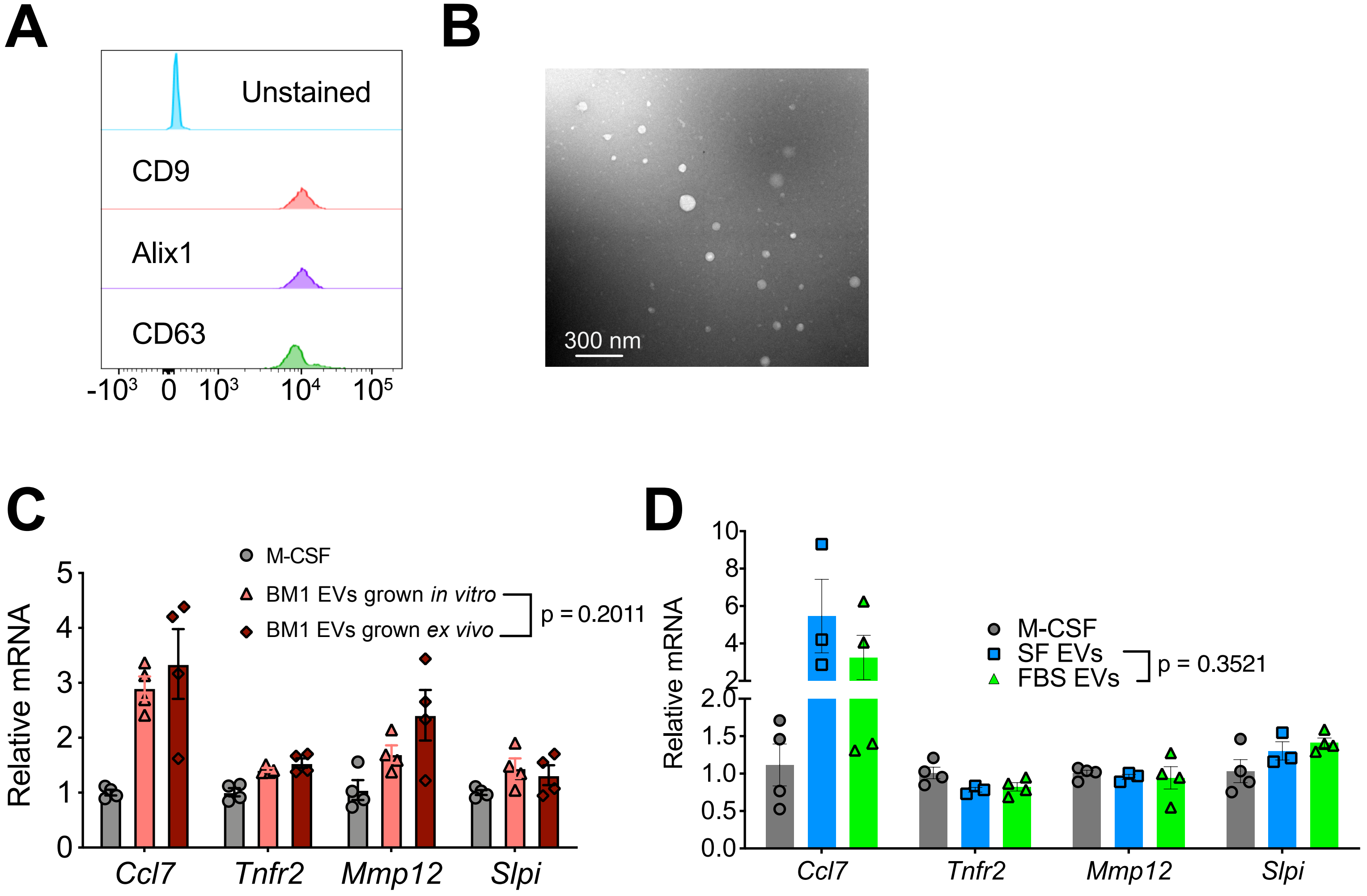

Figure S2: Validation of small-EV markers by flow, immunoblot, and scanning EM A) Flow cytometry of EVs isolated by qEV SEC columns for CD63, Alix1, and CD9. **B)** Scanning electron microscopy of EVs isolated from BM1 cells by qEV columns. **C)** qRT-PCR of *Ccl7*, *Tnfr2*, *Mmp12*, and *Slpi* in TEMs treated with EVs isolated from BM1 cells grown *in vitro* or BM1 cells grown *ex vivo* after isolating from tumors, showing no difference in TEM programing (p-value shown for a two-way ANOVA). **D)** qRT-PCR of *Ccl7*, *Grn*, and *Mmp12* in TEMs treated with EVs isolated from BM1 cells grown to confluence and then isolated for 24 hours in serum-free (SF) media or isolated over 72 hours while cells grew in media supplemented with 10% EV depleted FBS, showing no difference in TEM programming (p-value shown for a two-way ANOVA).
