# Supplementary material for: Tumor Extracellular Vesicles Regulate Macrophage-Driven Metastasis through CCL5
Source: Cancers (Basel). 2021 Jul 10;13(14):3459. doi: 10.3390/cancers13143459 (PMC8303898; doi:10.3390/cancers13143459)
Supplement: Supplementary file 1 [file cancers-13-03459-s001.zip › Figure S3.pdf]

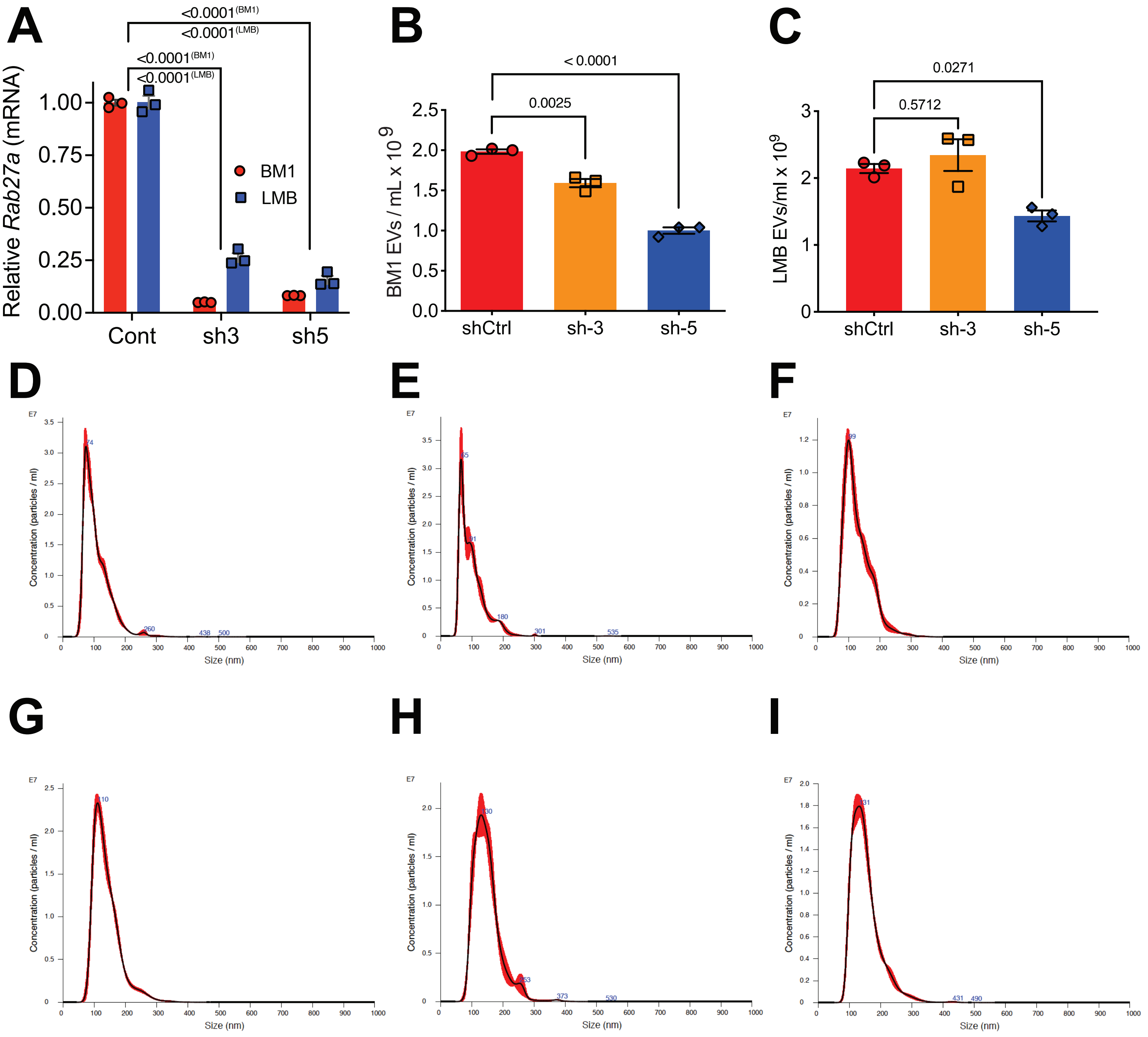

Figure S3: Reduction in EV secretion in BM1 and LMB cells upon Rab27a Knock-Down A) Mean +/- SEM of *Rab27a* in BM1 and LMB cells from vector control (Ctrl), shRab27a (sh-3), and shRab27a (sh-5). P-values calculated using a one-way ANOVA. **B)** Mean +/- SEM of EV concentration in BM1 cells from vector control (shCtrl, red), shRab27a (sh-3, orange), and shRab27a (sh-5, blue). P-values calculated using a one-way ANOVA. **C)** Mean +/- SEM of EV concentration in LMB shRab27a cells from shControl (black), sg-3 (orange), and sg-5 (blue). P-values calculated using a one-way ANOVA. **(D-F)** Represenative nanosight graphs of EV concentrations and size in BM1 shRab27a serum free cell media after a 2,000 x g spin and 220 um filter for each cell type expressing either shCtrl **(D)**, sh-3 **(E)**, or sh-5 **(F)**. **(G-I)** Represenative nanosight graphs of EV concentrations and size in LMB shRab27a cell serum free media after a 2,000 x g spin and 220 um filter for each cell type expressing either shCtrl **(G)**, sh-3 **(H)**, or sh-5 **(I)**.
