# Supplementary material for: Tumor Extracellular Vesicles Regulate Macrophage-Driven Metastasis through CCL5
Source: Cancers (Basel). 2021 Jul 10;13(14):3459. doi: 10.3390/cancers13143459 (PMC8303898; doi:10.3390/cancers13143459)
Supplement: Supplementary file 1 [file cancers-13-03459-s001.zip › Figure S4.pdf]

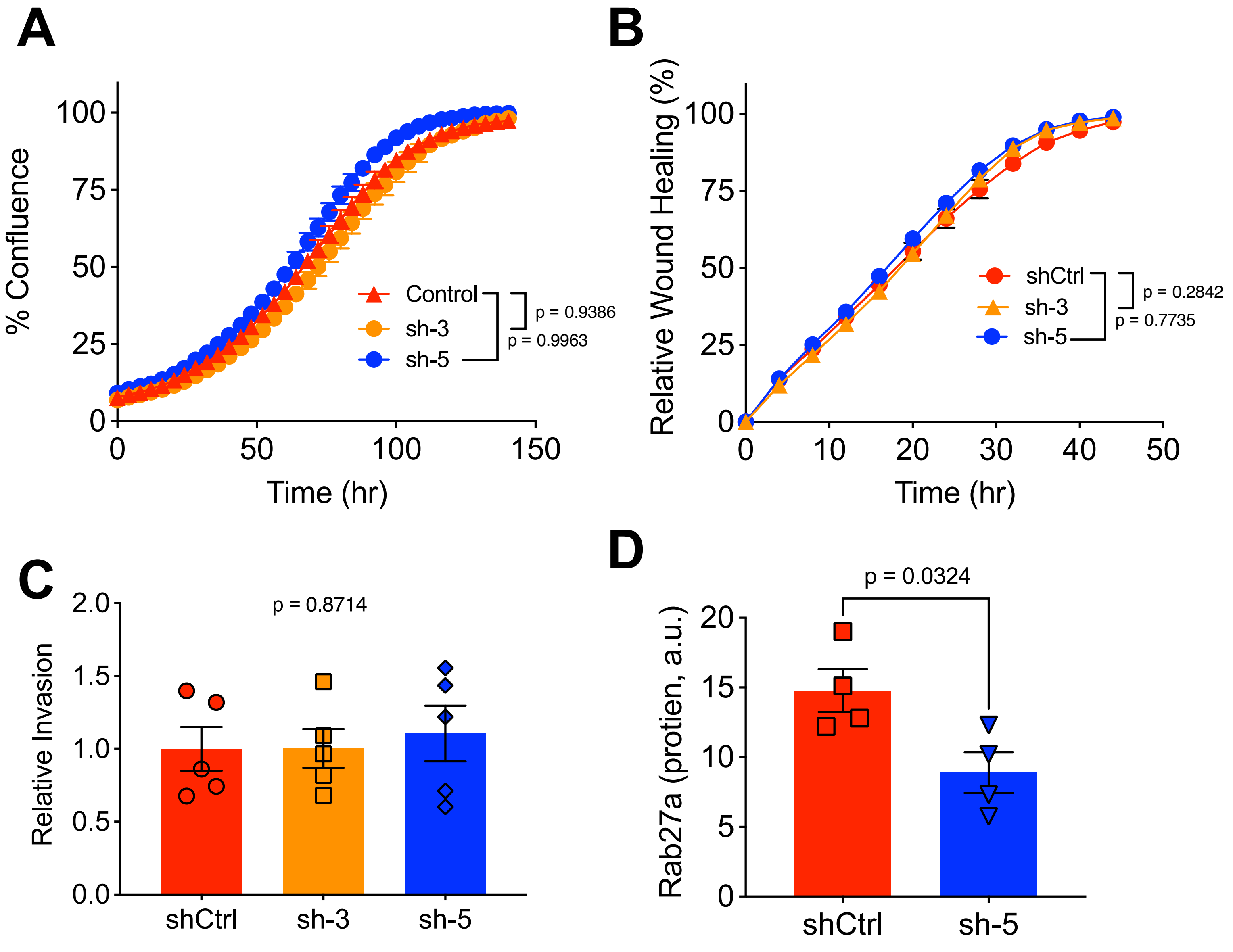

Figure S4: Rab27a Knock-Down in BM1 cells does not alter tumor growth, migration, or invasion A) Growth rates of BM1 shRab27a tumor cells. p-value shown for two-way ANOVA (N=16, individual points unavailable). **B)** Wound healing/migration in BM1 shRab27a cells. p-value shown for two-way ANOVA (N=16, individual points unavailable). **C)** Relative invasion in BM1 shRab27a tumor cells. p-value shown for one-way ANOVA between shCtrl, sh3, and sh5 (N=5). **D)** Rab27a levels normalized to tubulin in BM1 tumors from Control and shRab27a (N=4).
