# Supplementary material for: Tumor Extracellular Vesicles Regulate Macrophage-Driven Metastasis through CCL5
Source: Cancers (Basel). 2021 Jul 10;13(14):3459. doi: 10.3390/cancers13143459 (PMC8303898; doi:10.3390/cancers13143459)
Supplement: Supplementary file 1 [file cancers-13-03459-s001.zip › Figure S5.pdf]

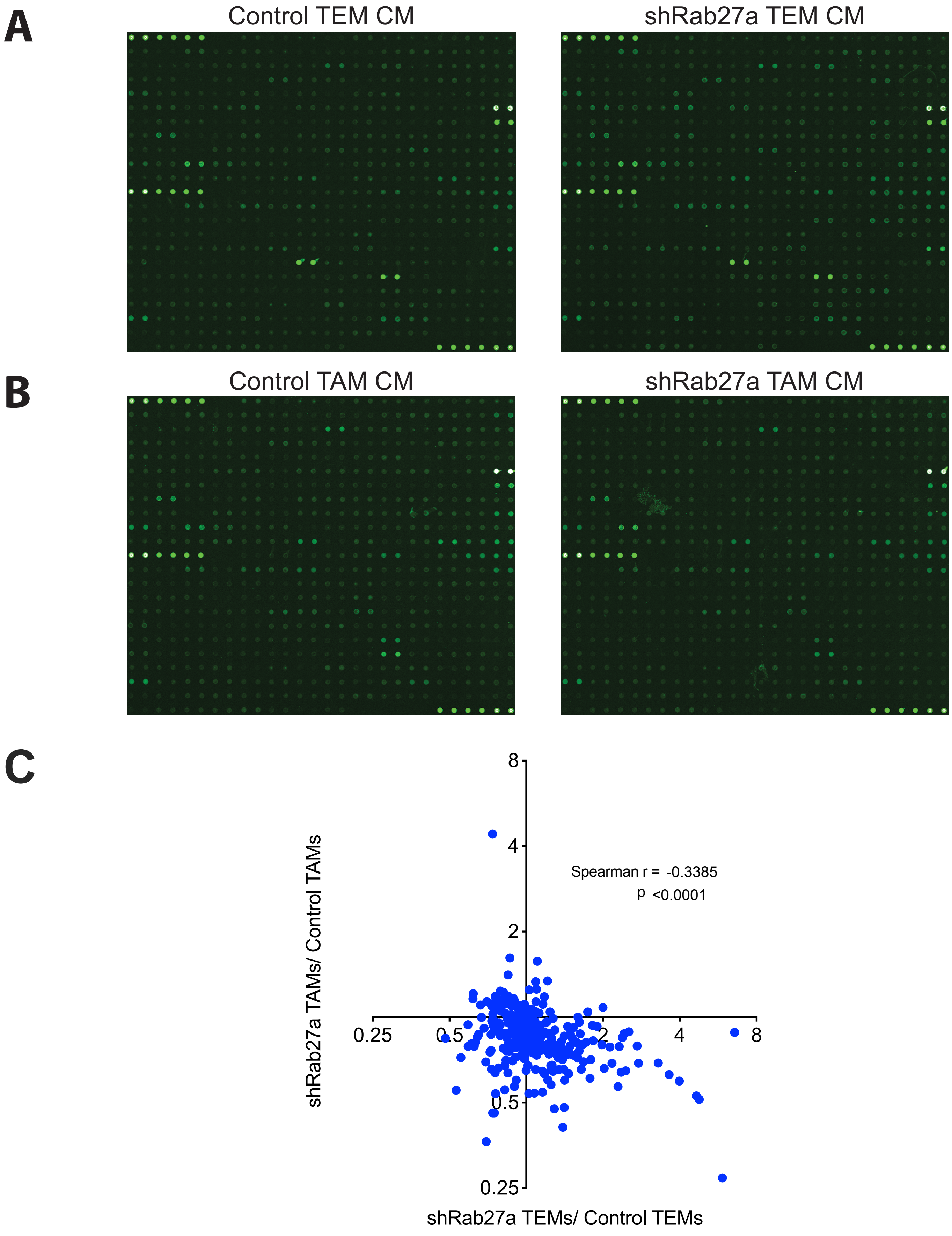

Figure S5: Cytokine array analysis of EV-programmed macrophages produced from control and shRab27a tumor cells (A-B) RayBiotech L308 cytokine array raw images for control and shRab EV programmed TEMs **(A)** and control and shRab TAM samples **(B)**. **(C)** Spearman correlations and corresponding p values shown for correlations between differential expression of shRab27a / Control TEMs to shRab27a / Control TAMs.
