# Supplementary material for: Tumor Extracellular Vesicles Regulate Macrophage-Driven Metastasis through CCL5
Source: Cancers (Basel). 2021 Jul 10;13(14):3459. doi: 10.3390/cancers13143459 (PMC8303898; doi:10.3390/cancers13143459)
Supplement: Supplementary file 1 [file cancers-13-03459-s001.zip › Figure S6.pdf]

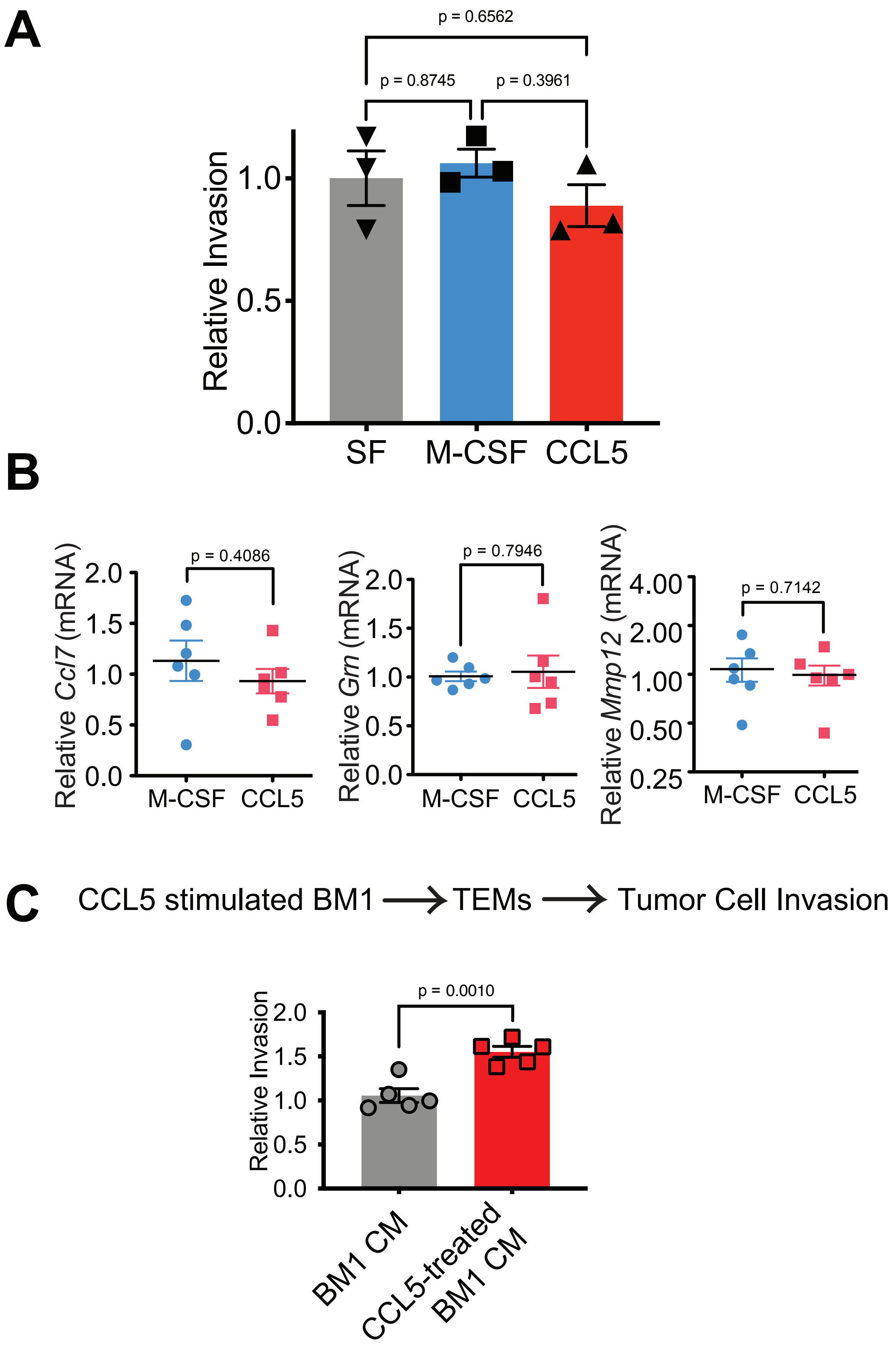

Figure S6: CCL5 protein alone not sufficient to drive macrophage programming A) Invasion of BM1 cells that were treated with SF media (SF), M-CSF derived TEM CM (M-CSF), or CCL5 derived TEM CM (CCL5). **B)** Relative gene epression of *Ccl7*, *Grn*, and *Mmp12* (normalized to *Gapdh*) in M-CSF vs CCL5 derived TEMs. **C)** Relative invasion of BM1 cells treated with TEM CM. TEMs were stimulated with BM1 CM or CM of BM1 cells that had previously been stimulated with CCL5 for 24 hrs. Relative invasion is compared against BM1 CM alone.
