# Supplementary material for: Tumor Extracellular Vesicles Regulate Macrophage-Driven Metastasis through CCL5
Source: Cancers (Basel). 2021 Jul 10;13(14):3459. doi: 10.3390/cancers13143459 (PMC8303898; doi:10.3390/cancers13143459)
Supplement: Supplementary file 1 [file cancers-13-03459-s001.zip › Figure S7.pdf]

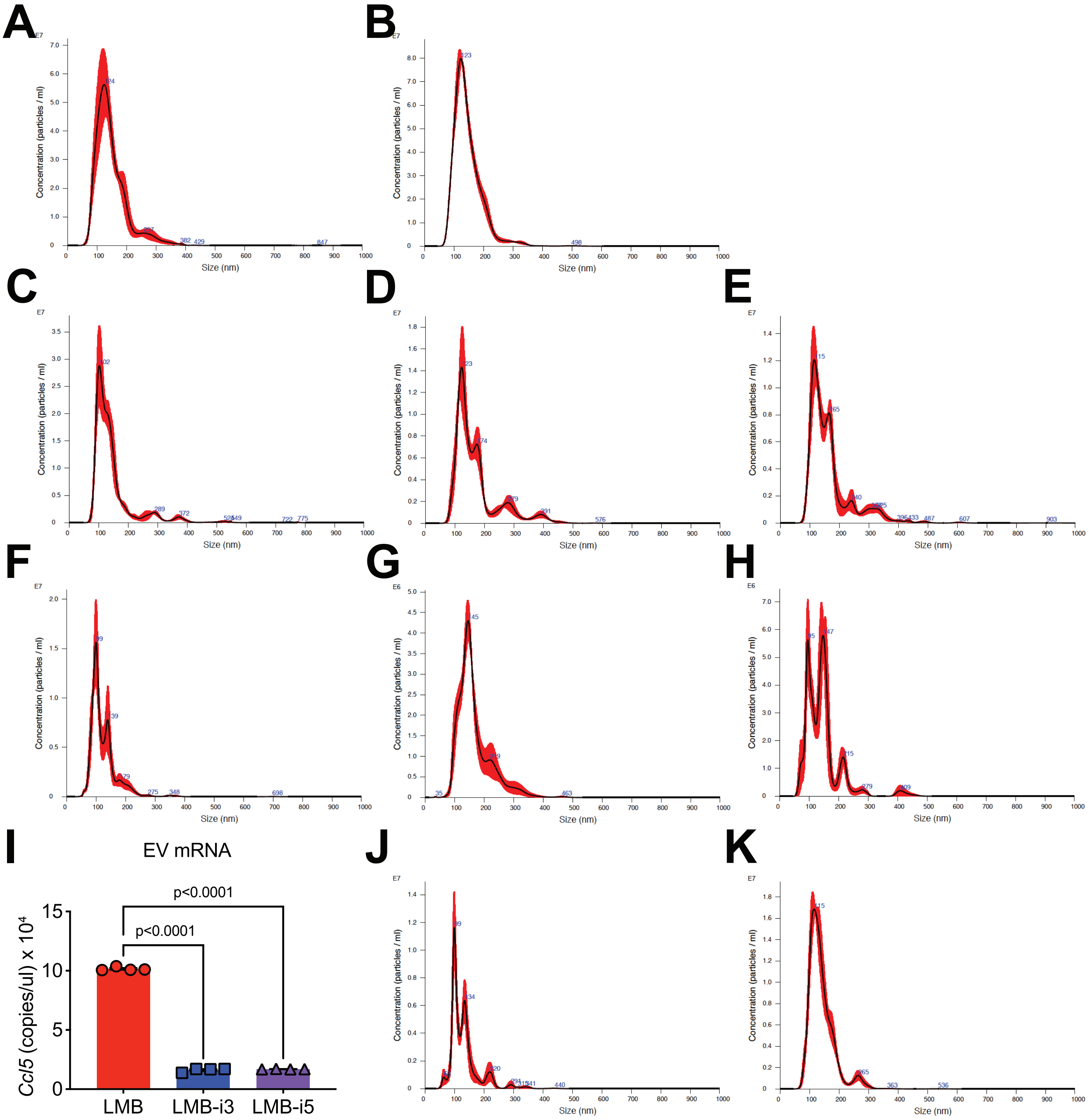

Figure S7: CCL5 regulates EV secretion in BM1 and LMB tumor cells (A-B) Representative nanosight graphs of EV concentrations and size in BM1 cell media after a 2,000 x g spin and 220 um filter for each cell type, control **(A)** and *CCL5* **(B)**. **(C-E)** Represenative nanosight graphs of EV concentrations and size in BM1 dCas9 cell media after a 2,000 x g spin and 220 um filter for each cell type, sg-control **(C)**, sg-*CCL5*-2 **(E)**, and sg-*CCL5*-3 **(E)**. **(F-H)** Represenative nanosight graphs of EV concentrations and size in LMB dCas9-KRAB cell media after a 2,000 x g spin and 220 um filter for each cell type, sg-control **(F)**, sg-*Ccl5*-i3 **(G)**, and sg-*Ccl5*-i5 **(H)**. **I)** *Ccl5* mRNA in LMB dCas9-KRAB cell EVs with sg-control (red), sg-*Ccl5*-i3 (blue), and sg-*Ccl5*-i5 (purple). **(J-K)** Represenative nanosight graphs of EV concentrations and size in LMB dCas9-VPR cell media after a 2,000 x g spin and 220 um filter for each cell type, sg-control **(J)** and sg-*Ccl5*-a5 **(K)**.
