# Supplementary material for: Tumor Extracellular Vesicles Regulate Macrophage-Driven Metastasis through CCL5
Source: Cancers (Basel). 2021 Jul 10;13(14):3459. doi: 10.3390/cancers13143459 (PMC8303898; doi:10.3390/cancers13143459)
Supplement: Supplementary file 1 [file cancers-13-03459-s001.zip › Figure S8.pdf]

A

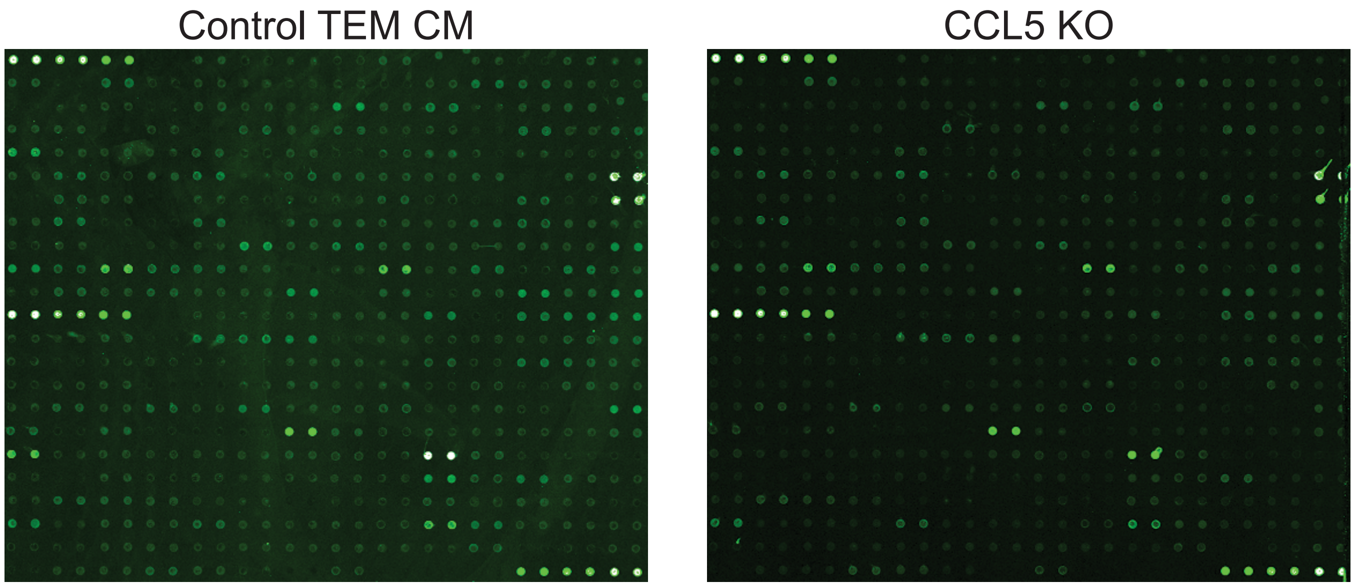

B

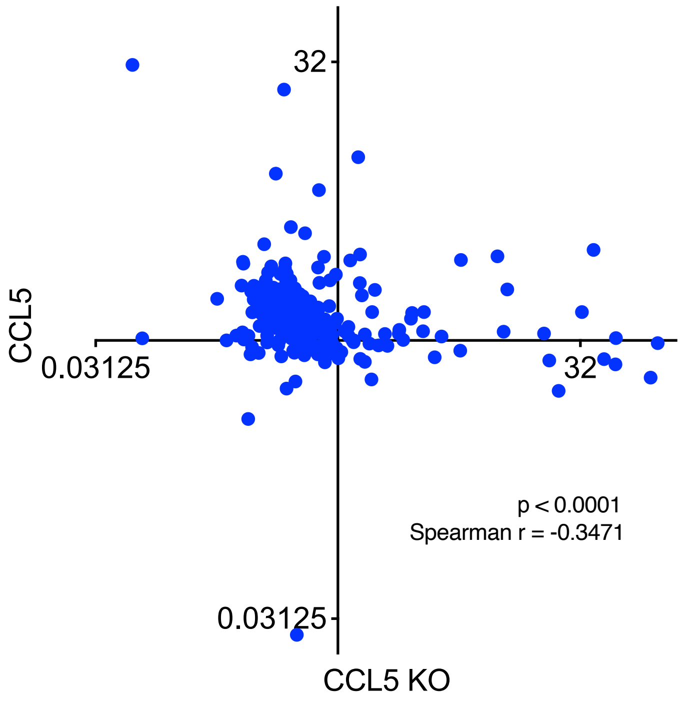

Figure S8: Cytokine array analysis of macrophages programmed with either control or CCL5-Knock-Down EVs (A) RayBiotech L308 cytokine array raw images for control and CCL5 KO EV programmed TEMs **(B)** Spearman correlations and corresponding p values shown for correlation between differential expression of CCL5 KO / Control TEMs to CCL5 / Control TAMs [previously published dataset]
