# Supplementary material for: Tumor Extracellular Vesicles Regulate Macrophage-Driven Metastasis through CCL5
Source: Cancers (Basel). 2021 Jul 10;13(14):3459. doi: 10.3390/cancers13143459 (PMC8303898; doi:10.3390/cancers13143459)
Supplement: Supplementary file 1 [file cancers-13-03459-s001.zip › Figure S9.pdf]

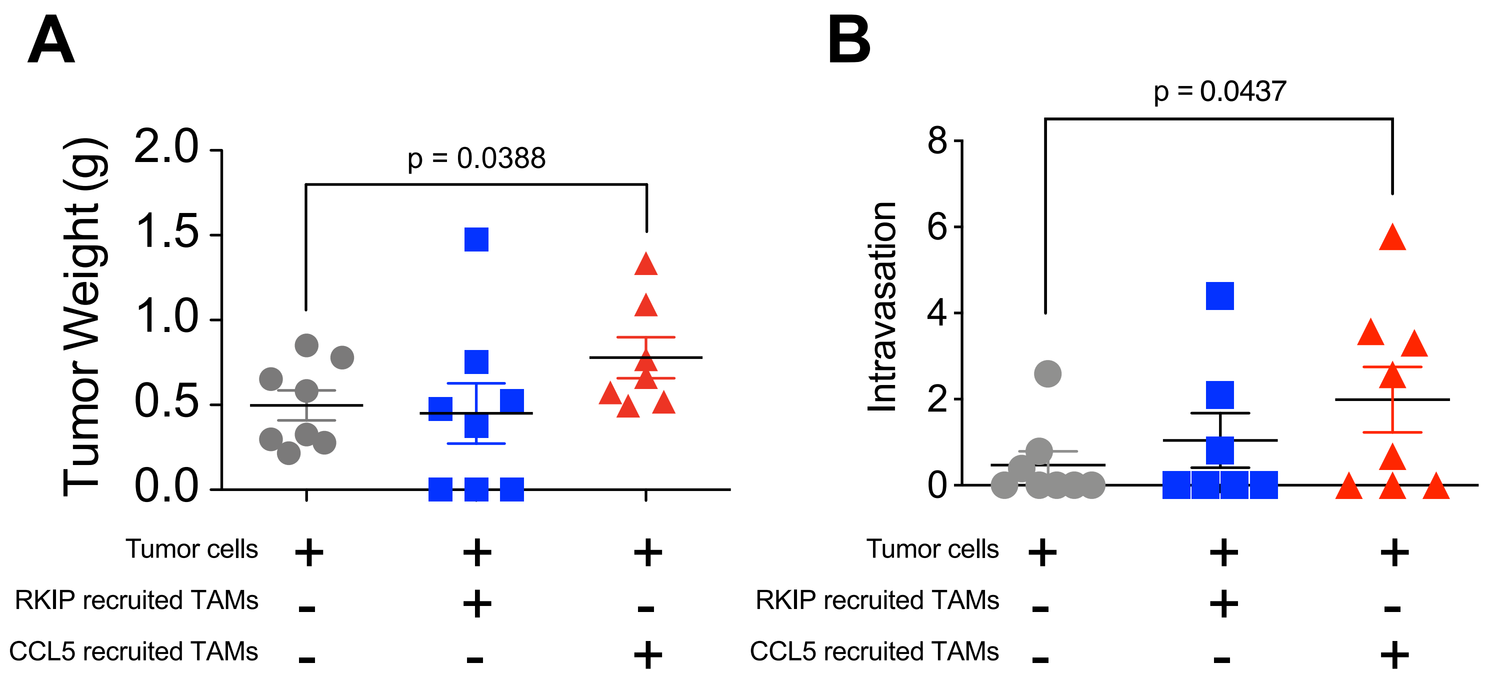

Figure S9: CCL5 recruited TAMs increase both tumor cell growth and metastasis Data shown are from tumors injected into the fatpad of athymic nude mice with or without TAMs from the denoted type of tumor. **A)** Final tumor weights between control tumors (N=8) and those co-injected with RKIP-derived (N=8) or CCL5-derived TAMs (N=7). **B)** Intravasation of tumor cells into the blood stream in control tumors (N=8) and those co-injected with RKIP-derived (N=7) or CCL5-derived TAMs (N=8).
