# Supplementary material for: Tumor Extracellular Vesicles Regulate Macrophage-Driven Metastasis through CCL5
Source: Cancers (Basel). 2021 Jul 10;13(14):3459. doi: 10.3390/cancers13143459 (PMC8303898; doi:10.3390/cancers13143459)
Supplement: Supplementary file 1 [file cancers-13-03459-s001.zip › Table S1.pdf]

	CCL5 KO / TEM	shRab27a (TAM)	Human Orthologue
**6Ckine_**	0.428	0.655374068	CCL21
**Activin_C**	0.366	0.630317579	INHBC
**AgRP**	0.543	0.72145277	AGRP
**ALCAM**	0.309	0.675318159	ALCAM
**AR_(Amphiregulin)**	0.545	0.648836338	AREG
**BCMA_/_TNFRSF17**	0.387	0.644563246	TNFRSF17
**CCR10**	0.444	0.677991393	CCR10
**CD11b**	0.472	0.655046439	ITGAM
**CD30_L**	0.388	0.713990936	TNFSF8
**CTACK_**	0.617	0.74534265	CCL27
**CXCR4**	0.326	0.746808333	CXCR4
**DR3_/_TNFRSF25**	0.397	0.674791433	TNFRSF25
**E-Selectin**	0.546	0.653453773	SELE
**Eotaxin_**	0.456	0.734390677	CCL11
**FAM3B**	0.461	0.59966362	FAM3B
**GDF-3**	0.619	0.636247242	GDF3
**GM-CSF**	0.482	0.734235283	CSF2
**IFN-alpha_/_beta_R2**	0.526	0.700998295	IFNAR2
**IFN-beta**	0.343	0.658557013	IFNB1
**IFN-gamma_**	0.397	0.741838408	IFNG
**IGFBP-2**	0.674	0.631671981	IGFBP2
**IL-1_alpha**	0.394	0.578353313	IL1A
**IL-1_R4_/_ST2**	0.597	0.717654175	IL1RL1
**IL-11**	0.658	0.695898858	IL11
**IL-12_p40/p70**	0.419	0.750468292	IL12
**IL-13**	0.618	0.479993906	IL13
**IL-16**	0.614	0.64691716	IL16
**IL-17**	0.406	0.741049881	IL17
**IL-21_R**	0.061	0.539361574	IL21R
**IL-27**	0.467	0.569365157	IL27
**IL-28_/_IFN-lambda**	0.605	0.708383954	IFNL1
**IL-31**	0.578	0.639991875	IL31
**IL-4_R**	0.374	0.7342391	IL4R
**IL-5**	0.439	0.633675102	IL5
**IL-5_R_alpha**	0.356	0.600459112	IL5RA
**IL-6**	0.368	0.598692437	IL6
**IL-7**	0.363	0.733081602	IL7
**LIGHT_/_TNFSF14**	0.259	0.459686472	TNFSF14
**LIX**	0.725	0.688491259	CXCL5
**LRP-6**	0.473	0.6452092	LRP6
**MCP-5**	0.605	0.655719424	CCL12
**MFG-E8**	0.203	0.705591042	MFGE8
**MIP-1_gamma**	0.257	0.608784351	CCL15
**MIP-2**	0.345	0.474949903	CXCL2
**MIP-3_beta_**	0.358	0.554783655	CCL19
**MMP-24_/_MT5-MMP**	0.293	0.538162659	MMP24
**Neuregulin-3_/_NRG3**	0.417	0.67419472	NRG3
**Osteopontin**	0.053	0.56993669	SPP1
**P-Selectin**	0.286	0.27138117	SELP
**PF-4**	0.750	0.627192038	PF4
**Prolactin**	0.515	0.527435165	PRL
**RELM_beta**	0.511	0.722290232	RETNLB
**SDF-1**	0.277	0.409840951	SDF1
**Serum_Amyloid_A1**	0.374	0.717918943	SAA1
**SLPI**	0.187	0.537423694	SLPI
**TGF-beta_3**	0.744	0.71999086	TGFB3
**TGF-beta_RII**	0.643	0.730471739	TGFBR2
**TLR1**	0.327	0.715817	TLR1
